# The emerging picture of the mitochondrial protein import complexes of Amoebozoa supergroup

**DOI:** 10.1186/s12864-017-4383-1

**Published:** 2017-12-29

**Authors:** Małgorzata Wojtkowska, Dorota Buczek, Yutaka Suzuki, Victoria Shabardina, Wojciech Makałowski, Hanna Kmita

**Affiliations:** 1 Laboratory of Bioenergetics, Institute of Molecular Biology and Biotechnology, Faculty of Biology, Adam Mickiewicz University, Umultowska 89, 61-614 Poznan, Poland; 2 Institute of Bioinformatics, Faculty of Medicine, University of Muenster, Niels Stensen Strasse 14, 48149 Muenster, Germany; 3 Department of Medical Genome Sciences, Graduate School of Frontier Sciences, University of Tokyo, Kashiwa, Chiba 277-8562 Japan

**Keywords:** Amoebozoa, Mitochondria, Mitosomes, Protein import, TIM22 complex, TIM23 complex, small Tims, MIA complex, PAM complex, OXA complex

## Abstract

**Background:**

The existence of mitochondria-related organelles (MROs) is proposed for eukaryotic organisms. The Amoebozoa includes some organisms that are known to have mitosomes but also organisms that have aerobic mitochondria. However, the mitochondrial protein apparatus of this supergroup remains largely unsampled, except for the mitochondrial outer membrane import complexes studied recently. Therefore, in this study we investigated the mitochondrial inner membrane and intermembrane space complexes, using the available genome and transcriptome sequences.

**Results:**

When compared with the canonical cognate complexes described for the yeast *Saccharomyces cerevisiae*, amoebozoans with aerobic mitochondria, display lower differences in the number of subunits predicted for these complexes than the mitochondrial outer membrane complexes, although the predicted subunits appear to display different levels of diversity in regard to phylogenetic position and isoform numbers. For the putative mitosome-bearing amoebozoans, the number of predicted subunits suggests the complex elimination distinctly more pronounced than in the case of the outer membrane ones.

**Conclusion:**

The results concern the problem of mitochondrial and mitosome protein import machinery structural variability and the reduction of their complexity within the currently defined supergroup of Amoebozoa. This results are crucial for better understanding of the Amoebozoa taxa of both biomedical and evolutionary importance.

**Electronic supplementary material:**

The online version of this article (doi:10.1186/s12864-017-4383-1) contains supplementary material, which is available to authorized users.

## Background

**Fig. 1:**
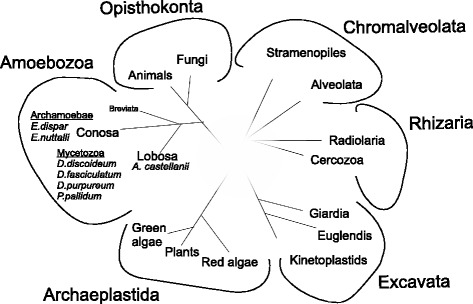
Schematic representation of the phylogenetic position of the studied organisms in the eukaryotic tree. Based on [1, 2, 46]

Currently, the division of eukaryotic organisms consists of six large supergroups, namely Chromalveolata, Excavata, Archaeplastida, Rhizaria, Amoebozoa, and Opisthokonta [1, 2]. As summarized in Fig. 1, the Amoebozoa, regarded as a sister clade to the Opisthokonta which involves animals and fungi, include some organisms that are known to have mitosomes, such as *Entamoeba histolytica*, an intestinal pathogen of humans [3, 4], but also organisms that have aerobic mitochondria, such as the slime mold *Dictyostelium discoideum*, a free-living organism that inhabits soil and compost [5], as well as the amoeba *Acanthamoeba castellanii*, a soil free-living amoeba also known as a facultative human parasite [6]. The organisms represent different subclades and subdivisions of the Amoebozoa [1]. *A. castellanii* represents the Lobosa, whereas *D. discoideum* and *E. histolytica* belong to the Conosa and are included in the Mycetozoa and Archamoebae, respectively (Fig. 1). Thus, the Amoebozoa encompass taxa of both biomedical and evolutionary importance, yet their genomic and transcriptomic diversity remains largely unsampled. The same applies to the mitochondrial protein import complexes. Accordingly, the data concerning the subunit organization of the mitochondrial protein import complexes for members of the same supergroup classified into distinct subclades and divisions are not well known.

**Fig. 2:**
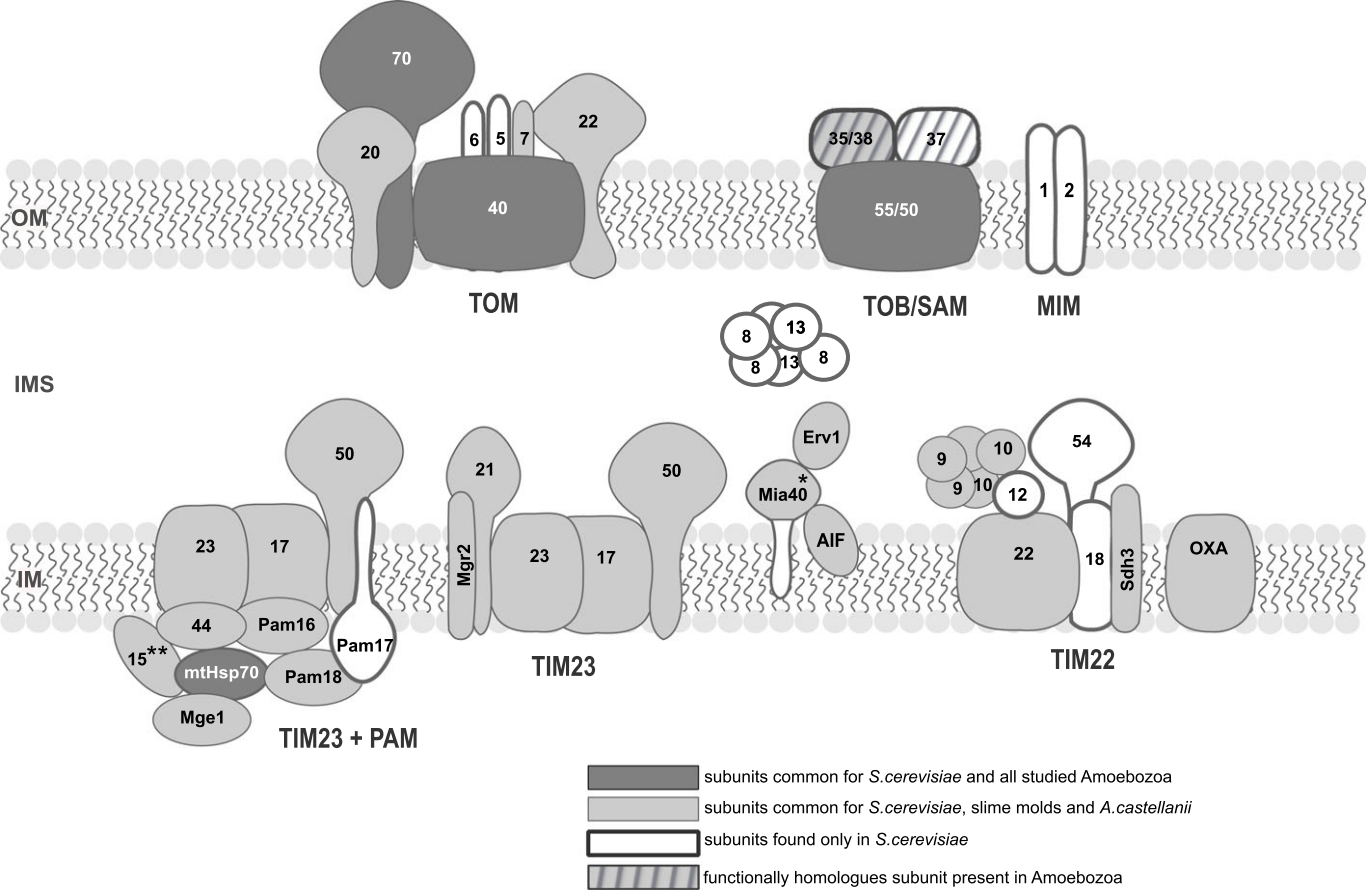
Schematic representation of the mitochondrial protein import complexes of *Saccharomyces cerevisiae* and their subunits shared with the studied amoebozoans. The TOM complex contains a channel-forming subunit (Tom40), receptors for precursor proteins (Tom20 and Tom70), an internal receptor, which also maintains the complex architecture (Tom22), and modulators of the complex assembly and stability (Tom5, Tom6, and Tom7). The TOB/SAM complex contains a channel-forming subunit (Tob55/Sam50), which cooperates with Sam35/Tob38 and Sam37/Mas37 in the recognition, transport, and integration of β-barrel proteins into the membrane. The TIM22 complex is composed of a channel-forming subunit (Tim22) and three modulators of the complex stability and activity, i.e. Tim18, Tim54, and Sdh3. The TIM23 complex consists of Tim50 (which functions as a receptor in the initial stages of precursor translocation), Tim23 and Tim17 (forming a channel within the complex), as well as Tim21 and Mgr2 (suggested to play an important role in the cooperation with the respiratory chain) The absence of Mgr2 enables the TIM23 complex interaction with the PAM complex. The complex consists of mtHsp70, Tim44, and a group of co-chaperones and supporting proteins, i.e. Pam16 (Tim16), Pam18 (Tim14), the nucleotide exchange factor Mge1, Pam17 and Tim15. The OXA complex is formed by Oxa1, which is thought to cooperate with its paralog, Oxa2, serving as an auxiliary subunit. The MIA complex consists of two proteins, namely the oxidoreductase Mia40 and sulfhydryl oxidase Erv1. *, subunits found only for slime molds; **, a subunit found only for *A. castellanii*; AIF – apoptosis inducing factor

Studies of the mitochondrial protein import machinery have revealed that the machinery consists of complexes located in all mitochondrial compartments and have provided information about their organization, function, and interplay. The most commonly used model organism in the studies is the yeast *Saccharomyces cerevisiae*. Consequently, the *S. cerevisiae* protein import machinery, schematically shown in Fig. 2, is defined as a canon and applied as a reference model in studies of the machinery of other eukaryotic organisms, including plants and animals [7–9]. The canon includes the presence of at least three complexes in the outer membrane, four in the inner membrane, and three in the intermembrane space. The TOM (translocase of the outer membrane), TOB/SAM (topogenesis of the mitochondrial outer membrane β-barrel proteins/sorting and assembly machinery) and MIM (mitochondrial import machinery) complexes are located in the outer membrane. The TOM complex represents a general entry gate for most proteins imported into mitochondria, whereas the TOB/SAM complex and MIM complex enable the insertion of β-barrel proteins and proteins containing one or more transmembrane-spanning helices into the membrane, respectively. The TIM22 (translocase of the inner membrane 22), TIM23 (translocase of the inner membrane 23), PAM (presequence translocase-associated motor), and OXA (oxidase assembly factor) complexes are located in the inner membrane. The TIM22 and TIM23 complexes mediate the import of precursor proteins with targeting signals located within their sequence and at their N-terminus, respectively. The PAM complex cooperates with the TIM23 complex to drive protein translocation into matrix, whereas the OXA complex participates in the insertion of proteins from the matrix side into the inner membrane. The MIA (mitochondrial intermembrane space assembly) complex as well as small Tim chaperone-like protein complexes (small Tims), i.e. Tim8-Tim13 and Tim9-Tim10-Tim12 complexes, are located in the intermembrane space. The MIA complex is responsible for the import of small intermembrane space proteins with the multiple cysteine residues due to the thiol-disulfide exchange, whereas small Tims associate with the TOB/SAM and TIM22 complexes to protect precursor proteins from misfolding in the intermembrane space.

Available data indicate that the subunit organization of the complexes of animals and plants is more or less similar to those depicted in Fig. 2 for *S. cerevisiae* (for reviews, see e.g. [7, 9–11] although some new subunits have recently been identified for animals e.g. [10] and plants [11]. Nevertheless, in other eukaryotes, the differences are more pronounced. Consequently, the commonly occurring subunits of the TOM, TOB/SAM, and TIM22 complexes are their channel-forming subunits, Tom40, Sam50/Tob55, and Tim22, respectively, whereas the common presence of Tim50, Tim17, and Tim23 is reported for the TIM23 complex, Erv1 for the MIA complex, and Tim9 and Tim10 for the small Tim protein complexes [12, 13]. In the case of the MIM and OXA complexes, data are still missing for this kind of analysis. The data concerning the protein import machinery are also strongly limited in mitosome-carrying organisms. As summarized by Heinz and Lithgow [12] as well as by Makiuchi and Nozaki [13], they are available for *Cryptosporidium parvum* (Chromalveolata), *Encephalitozoon cuniculi* (Opisthokonta), *Giardia intestinalis* (Excavata), and *E. histolytica* (Amoebozoa). The organisms carry Tom40 and Sam50/Tob55, although the latter was not detected for *G. intestinalis*, and only two receptor subunits of the TOM complex, Tom70 and Tom60, were predicted for *E. cuniculi* and *E. histolytica,* respectively. Moreover, *C. parvum* and *E. cuniculi* have Tim17 and Tim23 (the TIM23 complex) as well as Tim22 (the TIM22 complex), but it is difficult to distinguish the proteins at the level of their sequences. Other subunits of the complexes, Tim50 (the TIM23 complex) and Tim18 (the TIM22 complex), were found for *C. parvum* and *E. cuniculi* with the exception of *G. intestinalis* Tim18. In the case of small Tims, only Tim8 and Tim13 seem to be expressed by *C. parvum*. As far as the PAM complex subunits are concerned, mtHsp70 was found for all the mentioned organisms, Mge1 for *C. parvum*, *G. intestinalis*, and *E. histolytica*, while Tim44 and Pam16 were detected only for *C. parvum*.

Our previous study, based on searching of the available genome and transcriptome data concerning amoebozoan subunits of the TOM and TOB/SAM complexes, indicated that the complexes may display structural variability in the Amoebozoa lineage and their subunit number reduction, as compared with the corresponding canonical complexes of *S. cerevisiae* [14]. Accordingly, the predicted number of subunits of the TOM and TOB/SAM complexes were different for *A. castellanii*, and representatives of slime molds and entamoebas as well as within the slime molds and entamoebas. Importantly, entamoebas are proposed to contain mitosomes and *E. dispar*, and *E. nuttalii* are now recognized as separate species from the well-known pathogenic *E. histolytica* and *E. invadens*, although their pathogenicity is still a matter of debate [15–17]. To study the issue further, we extended our research on the transcriptome and genome analysis of the complexes located in the inner membrane and intermembrane space of the amoebozoan mitochondria. As mentioned above, they include the TIM22, TIM23, PAM, and OXA complexes located in the inner membrane as well as the MIA complex and the complexes of small Tim proteins forming Tim8-Tim13, and Tim9-Tim10-Tim12 complexes, all located in the intermembrane space. The predicted subunits indicate that for amoebozoans with aerobic mitochondria, organization variability of the complexes may be less pronounced than in the case of the mitochondrial outer membrane complexes. Nevertheless, the subunits predicted for the inner membrane and the intermembrane space complexes display different levels of diversity in regard to phylogenetic position and some of them have isoforms. Moreover, elimination of the complexes in the putative mitosome-bearing amoebozoans is predicted to be much more conspicuous than in the case of the outer membrane complexes. The results appear to be important for discussion on the complex localization and functions as well as their contribution to the protein import and functions of mitochondrial membranes.

## Methods

### Species of amoebozoans

The studied organisms included *Acanthamoeba castellanii* (Lobosa; Discosea), *Dictyostelium discoideum, D. purpureum, D. fasciculatum*, and *Polysphondylium pallidum* (Conosa; Mycetozoa), as well as *Entamoeba dispar* and *E. nuttalli* (Conosa; Archamoebae). Additional file 1: Table S1 summarizes data concerning their genome and transcriptome sequences.

*Acanthamoeba castellanii* strain Neff used in this study was delivered from American type Culture Collection (ATCC) with the number 30010.

### *Acanthamoeba castellanii* cell culture and isolation of total RNA

Cells of *Acanthamoeba castellanii* (strain Neff) were cultured at 28 °C, in an axenic environment in the standard medium described by Neff [18] with some modifications: 1.5% proteoso-peptone, 0.15% yeast extract, 30 mM MgCl_2_, 30 mM FeSO_4_, 27 mM CaCl_2_, 1.5% glucose, 2.5 mg/l vitamin B12, 1 mg/l vitamin B1, and 0.2 mg/l vitamin H. Cells in the trophozoite stage were collected in the intermediary phase after 48 h and next were frozen in liquid nitrogen and homogenized in TRizol reagent (Invitrogen). Total RNA was isolated according to the manufacturer’s instructions (Invitrogen). DNase I was added to eliminate remaining genomic DNA. The absence of DNA was confirmed by PCR and agarose gel electrophoresis.

### *Acanthamoeba castellanii* cDNA preparation, sequencing, and transcriptome assembly

cDNA was prepared using an mRNA-Seq Sample preparation Kit (Illumina), according to the manufacturer’s instructions. Sequencing of the cDNA, i.e. mRNA-Seq of *A. castellanii* was performed on the HiSeq 2000 platform (Illumina) with 36-bp single-end reads. AC_RNASeq data are available in DDBJ database; the accession number DRA006231 (BioSample number SAMD00097225). The obtained raw reads were subjected to quality control analysis. After removal of poor-quality sequences, short reads were assembled using Trinity RNA-Seq [19] with -SS_lib_type F (AC_RNASeq) and min_contig_length 300.

### Prediction of proteins

To find the best annotated amino acid sequences for subunits of the TIM22, TIM23, PAM, OXA, small Tims and MIA complexes, keyword searches against the NCBI (http://www.ncbi.nlm.nih.gov/) and Pfam (http://pfam.sanger.ac.uk) databases were performed. First, sets of well-known sequences from different species representing various eukaryotic lineages including subunits identified for *S. cerevisiae* as well as detected specifically for plant or animal mitochondrial protein import complexes (Additional file 1: Table S2) were used as queries in tBLASTn searches [20] against the transcriptome of *A. castellanii* with variable *e*-values (from 10^−3^ to 1). For the proteins that were not predicted by tBLASTn, a HMMER search based on Hidden Markov Model was performed [21]. In the case of multiple-protein hits, sequences giving the highest coverage of transcripts were selected. To translate the transcripts into protein sequences, the ExPASY server was used [22]. The amino acid sequences of putative proteins were subjected to a BLASTp [20] search in order to compare the sequences with available protein datasets of *A. castellanii* [6]. To find previously un-annotated proteins, a tBLASTn search against the available genome of *A. castellanii* was performed.

Subsequently, proteins predicted for *A. castellanii* were used in a tBLASTn search against the protein datasets of *Dictyostelium purpureum, D. discoideum, D. fasciculatum, Polysphondylium pallidum, Entamoeba dispar*, and *Entamoeba nuttalli* (Additional file 1: Table S1). For the proteins that were not predicted by the analysis, the tBLASTn algorithm was used against the available genomes of *D. discoideum*, *D. purpureum, D. fasciculatum, P. pallidum, E. dispar*, and *E. nuttalii.* Finally, reference sequences from various eukaryotic lineages (Additional file 1: Table S2) were used to predict proteins not found by the previously applied methods.

### Phylogenetic inference

Protein sequences were aligned using CLUSTAL W [23] and phylogenetic trees were inferred using the Neighbor-Joining method [24] with the amino acid substitution model using Poisson correction as implemented in MEGA (version 7) software [25, 26]. The data were bootstrapped by 1000 replicates [27].

## Results

We investigated the presence of genes encoding subunits of the mitochondrial protein import complexes located in the intermembrane space and inner membrane using available genome and transcriptome sequences. As summarized in Table S1 (Additional file 1), among the studied amoebozoans the transcriptome data are not available for the slime mold *Dictyostelium fasciculatum* and both the entamoebas, i.e. *Entamoeba nuttalli* and *Entamoeba dispar*. Moreover, in the case of the amoeba *Acathamoeba castellanii* we applied two transcriptome datasets, one already available at GenBank [6] and the second (AC_RNAseq) assembled by us (see Methods). The reason is that we noticed some differences between the transcriptome datasets reflected in amino acid sequences of proteins predicted for the mitochondrial outer membrane [14]. The involvement of transcriptome data supplies more information regarding gene isoforms and gene structure giving more opportunity in prediction process of examined genes.

### Subunits predicted for *A. castellanii*

The application of the available GenBank and AC_RNASeq enabled prediction of 17 *A. castellanii* possible subunits of mitochondrial protein import complexes located in the intermembrane space and the inner membrane (Table 1; for the complex organization see Fig. 2). Importantly, four of the subunits appeared to have isoforms (Tim9, Tim10, Pam16, and Pam18). All the predicted proteins displayed a high level of amino acid sequence identity to the GenBank data but also some differences were observed when AC_RNASeq was taken into account. The differences are shown in Additional file 1: Figures S1 and S2.

**Table 1:** The predicted subunits of Tim9-10-12, TIM22, TIM23 complexes of the studied Amoebozoa

Subunit	Tim9	Tim10	Tim22	Sdh3	Tim17	Tim21	Tim23	Tim50	Mgr2	
*A.castellanii*	A: XP_004340603 MF045879 B:MF045869 C: XP_ 004340763 MF045880	A: XP_004339376 MF045875 B: XP_004336134 MF045868	AAT66174 MF045866	XP_ 004367486 MF045878	XP_ 004347782 MF045865	XP_004340873	XP_004336406 MF045876	XP_004344840 MF045867	XP_004333275 MF045874	
*D.discoideum*	XP_644845	XP_639927	XP_638851	XP_643757	XP_637129	XP_629896	XP_637678	XP_646611	XP_637862	
*D.fasciculatum*	XP_004354109	XP_004358579	XP_004361430	XP_004362434	XP_00435582	XP_004350055	XP_004361430	XP_004354426	XP_004354259	
*D.purpureum*	XP_003293366	XP_003286712	XP_003289095	XP_003290414	XP_003289186	XP_003287691	XP_003287004	XP_003284507	XP_003284359	
*P.pallidum*	EFA81367	EFA78336	EFA79918	EFA81038	EFA79540	EFA79679	EFA84293	EFA78450	EFA75083	
*E.dispar/nuttalli*										
Subunit	Pam16	Pam18	Tim44	mtHsp70	Tim15	Mge1	Mia40	AIF	Erv1	Oxa1
*A.castellanii*	A: XP_ 004335678 MF045873 B: XP_ 004368030 MF045872	A: XP_ 004345808 MF084995 B: MF045881	XP_004348518.1	XP_004339232 MF045871	XP_004335035.1	MF045870			XP_004367724	XP_004344684.1
*D.discoideum*	Q54SV6	Q54QN1	EAL64247	Q8I0H7		XP_638912	XP_643501	XP_636815.1	XP_645302	A:EAL66882.2 B: EAL64905.1
*D.fasciculatum*	XP_004356346	EGG24887	XP_004367372	XP_004360938		XP_004362148	XP_004357088	XP_004357359.1	XP_004360441	A:EGG21561.1 B: EGG16308.1
*D.purpureum*	XP_003293468	EGC31346	XP_003293823	XP_003283943		XP_003294616	XP_003291222	EGC30677.1	XP_003286950	A:XP_003292615.1 B: XP_003287360.1
*P.pallidum*	EFA78034	EFA86354	EFA81857	EFA85858		EFA77076	EFA83431	XP_020436302.1	EFA83584	A: EFA84401.1 B: EFA75517.1
*E.dispar*				XP_001736890						
*E.nuttalli*				XP_008855870						

Underlined letters denote sequences determined in this study Not-underlined letters denote sequences stored in the GenBank as specifically annotated proteins

**Fig. 3:**
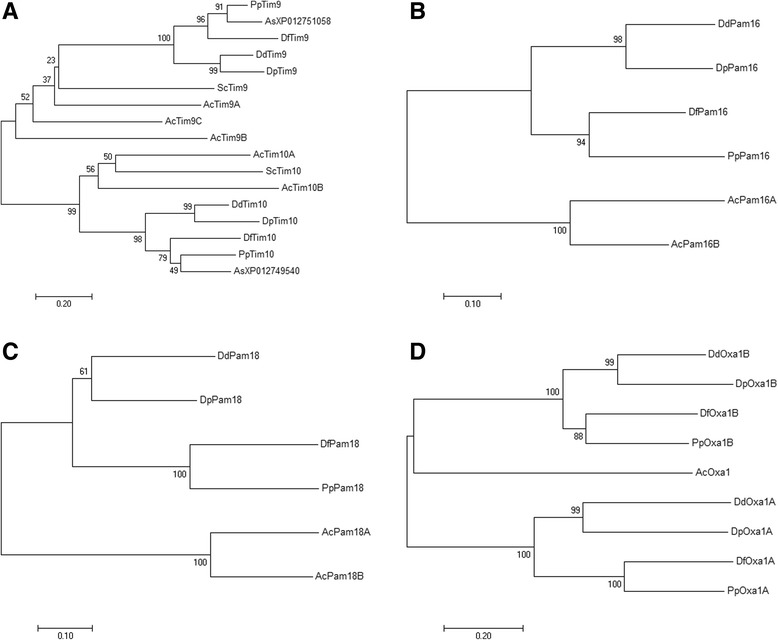
Phylogenetic relationships of the isoforms predicted for Tim9, Tim10, Pam16, Pam18 and Oxa1, based on unrooted phylogenetic trees constructed using the Neighbor-Joining method. The data were bootstrapped by 1000 replicates. All figures show unrooted trees. **a** Tim9 andTim10; **b** Pam16; **c** Pam18; **d** Oxa1. Ac – *Acanthamoeba castellanii*; Dd – *Dictyostelium discoideum*; Dp – *D. purpureum*; Df – *D. fasciculatum*; Pp – *Polysphondylium pallidum*; Sc – *Saccharomyces cerevisiae*; As – *Acytostelium subglobosum* (Amoebozoa)

The amino acid sequences predicted for Tim21, Tim44, Erv1 and Oxa1 have been already specifically annotated so the existing accession numbers were added to Table 1. The sequences predicted for Tim9C, Tim10A, Sdh3, Tim17, Tim23, Tim15 and Pam18A have been named specifically (Table 1, underlined letters; Tim15 is under the process of annotation). To check the accuracy of the predicted *A. castellanii* Tim9 and Tim10 isoforms the phylogenetic analysis was performed (Fig. 3a). In the obtained phylogenetic tree, two clusters representing Tim9 and Tim10 families can be easily distinguished. Although bootstrap values were not very high for the internal branches within two clusters, they were separated by the middle branch, which was supported by the bootstrap value of 98%. Thus, the isoforms were predicted properly and they appeared to be orthologs. Accordingly, to estimate amino acid similarity between *A. castellanii* isoforms of Pam16 and Pam18 and their phylogenetic relationships, phylogenetic trees were built (Fig. 3b–c). The obtained results indicated that the slime mold clades of Pam16 and Pam18 grouped with *A. castellanii* clades of these proteins. Thus the *A. castellanii* proteins appeared to be orthologs.

For the rest of the predicted proteins some differences were observed between AC_RNAseq and the GenBank data (Additional file 1: Figure S1). Consequently, we proposed some corrections of sequences predicted for Tim9A, Tim10B, Tim22, Tim50, Mgr2, Pam16A, Pam16B, and mtHsp70. In the case of the predicted Tim9A, the protein already stored in the GenBank (XP_004340603) was mis-annotated, as it contained only part of the functional domain of Tim9 proteins, and was fused with EF-hand Ca^2+^-binding domain that was removed from the corrected Tim9A version. The predicted Tim10B, Tim22, and Pam16A were longer than cognate annotated proteins (XP_004336134, AAT66174 and XP_004335678, respectively), whereas Tim50 and Mgr2 were longer than hypothetical proteins (XP_004344840 and XP_004333275, respectively). The protein XP_004368030.1 appeared to be encoded by an erroneously fused gene that was eliminated from the corrected Pam16B version. In the case of predicted mtHsp70, the correction concerned two amino acid substitutions at the C-terminus, namely alanine to serine and leucyne to methionine. Finally, the genes encoding Tim9B, Pam18B, and Mge1 were not present in the GenBank data but were supported by hits found in tBLASTn alignment against AC_RNASeq (Additional file 1: Figure S2).

Summing up, as can be concluded from data shown in Table 1, organization of the *A. castellanii* TIM23 and PAM complexes may be identical to cognate complexes of *S. cerevisiae* whereas in the case of small Tims, TIM22, OXA and MIA complexes some subunits described for cognate *S. cerevisiae* complexes appear not to be present.

### Subunits predicted for slime molds

Altogether 18 subunits were predicted for the intermembrane space and inner membrane import complexes (Fig. 2) of the studied slime molds, i.e. *D. discoideum, D. fasciculatum, D. purpureum* and *Polysphondylium pallidum* (Table 1). Most of the proteins were identical to sequences stored in the GenBank as specifically annotated or hypothetical proteins (the latters are marked as underlined letters in Table 1). The isoforms were predicted only for Oxa1. To check their accuracy phylogenetic analysis was performed and the obtained phylogenetic tree supported the notion that the slime mold Oxa1A and Oxa1B were paralogs (Fig. 3d). Moreover, for *A. castellanii* Oxa1 a higher similarity to the slime mold Oxa1B was observed. The only correction concerned *P. pallidum* Tim9, detected by the genome data analysis. The sequence turned out to be a part of a sequence stored in the GenBank under accession number EFA81367 (Additional file 1: Figure S1B).

Summing up, it appears that organization of the slime mold TIM23, PAM and MIA complexes may be identical to cognate complexes of *S. cerevisiae* whereas in the case of small Tims, TIM22 and OXA complexes some subunits are probably not present. Interestingly, with exception of Mia40, the missing subunits are the same as for *A. castellanii*.

### Subunits predicted for entamoebas

The only subunit predicted for *E. dispar* and *E. nuttalli* was mtHsp70. The proteins have been already annotated (Table 1).

### Phylogenetic position of the predicted proteins

To assess the phylogenetic position of the predicted proteins we estimated the similarity of their amino acid sequences to cognate subunits (Table 2). The predicted subunits displayed mostly the highest similarity to cognate proteins of Opisthokonta (fungi and animals) although the highest similarity to Archaeplastida (plants), Chromalveolata, and Excavata proteins was also observed in some cases. The least diverse in terms of similarity to reference proteins was the MIA complex, as the predicted subunits displayed the highest similarity to Opisthokonta proteins (animals and fungi) with the exception of *D. fasciculatum* Mia40 (Archaeplastida, plants). The prevailing similarity of the predicted subunits to Opisthokonta proteins (animals and fungi) was also observed for TIM23 complex subunits as the similarity to Archaeplastida proteins was only observed for the predicted *A. castellanii* Tim23 and *P. pallidum* Tim21. In the case of TIM22 complex as well as Tim9 and Tim10 proteins, the majority of the predicted proteins also displayed the highest similarity to the cognate Opisthokonta proteins (animals and fungi). However, the highest similarity to Chromalveolata cognate proteins was observed for the predicted *D. fasciculatum* Sdh3 as well as *A. castellanii* Tim9C and the slime mold Tim9 with exception of *P. pallidum* Tim9, which appeared to be the most similar to the Archaeplastida cognate proteins.

**Table 2:** The highest similarity of the amino acid sequences of the predicted subunits of Tim9-10-12, TIM22, TIM23, PAM, MIA, and OXA complexes to the applied reference sequences

Subunit	Tim9	Tim10	Tim22	Sdh3	Tim17	Tim21	Tim23	Tim50	Mgr2	
*A.castellanii*	A: *C. neoformans* P0CR97 Opisthokonta (Fungi) 9.00E-18 B: *C.neoformans* P0CR97 Opisthokonta (Fungi) 1.00E-15 C: *G.theta* CAJ736551 Chromalveolata 9.00E-16	A: *R. pulchellus* JAA56716 Opisthokonta (Animalia) 5.00E-18 B: *H.sapiens* AAH32133 Opisthokonta (Animalia) 5.00E-18	*S.canaria* XP_009093020 Opisthokonta (Animalia) 2.00E-32	*H.sapiens* AAH20808 Opisthokonta (Animalia) 8.00E-29	*A.melifera* XP_003249498 Opisthokonta (Animalia) 5.00E-47	*H.glaber* XP_004889998 Opisthokonta (Animalia) 6.00E-22	*M. truncatula* XP_003591008 Archaeplastida (Plantae) 1.00E 23	*U. maydis* Q4PEW9 Opisthokonta (Fungi) 5.00E-32	*L gigantea* ESO93582 Opisthokonta (Animalia) 1.00E-19	
*D.discoideum*	*P.reichenovi* CD966465 Chromalveolata 6.00E-13	*C.neoformans* P0CR99 Opisthokonta (Fungi) 3.00E-19	*N.parvum* XP_007579699 Opisthokonta (Fungi) 2.00E-24	*H.sapiens* AAH20808 Opisthokonta (Animalia)1.00E-26	*C.lusitaniae* EEQ38478 Opisthokonta (Fungi) 1.00E-36	*B.dorsalis* JAC53599 Opisthokontastokhonta (Animalia) 2.00E- 08	*D.reiro* NP_001099068 Opisthokonta (Animalia) 9.00E-11	*U. maydis* Q4PEW9 Opisthokonta (Fungi) 5.00E-100	*A.queenslandica* XP_003384516 Opisthokonta (Animalia) 2.00E-11	
*D.fasciculatum*	*G.theta* CAJ73651 Excavatea 2.00E-12	*T.minima* EON98536 Opisthokonta (Fungi) 7.00E-12	*C.glabrata* Q6FT37 Opisthokonta (Fungi) 5.00E-27	*N.gaditana* EWM21144 Chromaleolata 1.00E-18	*U.reesii* EEP81326 Opisthokonta (Fungi) 2.00E-39	*M.nubicus* XP_0089444459 Opisthokonta (Animalia) 4.00E-05	*D.reiro* NP_001099068 Opisthokonta (Animalia) 2.00E-11	*S.pombe* CAA17836 Opisthokonta (Fungi) 1.00E-18	*A.kawachii IFO* GAA85302 Opisthokonta (Fungi) 1.00E-18	
*D.purpureum*	*P.reichenovi* CD066465 Chromalveolata 1.00E-12	*S.borealis* EZ91432 Opisthokonta (Fungi) 1.00E-18	*N.parvum* XP_007579699 Opisthokonta (Fungi) 3.00E-26	*H.sapiens* AAH20808 Opisthokonta (Animalia) 2.00E-25	*M.hirsutus* ABM55650 Opisthokonta (Animalia) 6.00E-38	*J.jaculus* XP_004654873 Opisthokonta (Animalia) 1.00E-04	*D.reiro* NP_001099068 Opisthokonta (Animalia) 5.00E-11	*T.tonsurans* EGD98204 Opisthokonta (Fungi) 2.00E-20	*E. granulosus* EUB54634 Opisthokonta (Animalia) 8.00E-16	
*P.pallidum*	*B.distachon* XP_003578989 Archaeplastida (Plantae) 3.00E-14	*N.crassa* AF343076 Opisthokonta (Fungi) 1.00E-17	*C.glabrata* Q6FT37 Opisthokonta (Fungi) 1.00E-32	*H.sapiens* AAH20808 Opisthokonta (Animalia) 7.00E-23	*M.hirsutus* ABM55650 Opisthokonta (Animalia) 9.00E-42	*Ch.reinhardti* EDP00063 Archaeplastida (Plantae) 5.00E-04	*D.reiro* NP_001099068 Opisthokonta (Animalia) 5.00E-12	*U.maydis* Q4PEW9 Opisthokonta (Fungi) 2.00E-21	*S.japonicus* EEB06081 Opisthokonta (Fungi) 2.00E-07	
*E.dispar/nuttalli*										
Subunit	Pam16	Pam18	Tim44	mtHsp70	Tim15	Mge1	Mia40	AIF	Erv1	Oxa1
A.castellanii	A:*P.lutzi* EEH38935 Opisthokonta (Fungi) 2.00E-27 B: *P.requeforti* CMD30944 Opisthokonta (Fungi) 3.00E-29	A: *Ch. Reinch*. EDP02285 Archaeplastida (Plantae) 2.00E-33 B: *O.hannah* ETE61117 Opisthokonta (Animalia) 1.00E-32	*R. solani* ELU41216 Opisthokonta (Fungi) 2.00E-10	*Blastocystis *sp. AFL03352 Chromaleolata 0.0	*C.cardunculus* KVI05012.1 Archaeplastida (Plantae) 7.00E-22	*E.huxlei* EOD12618 Chromaleolata 5.00E-52			*S.cerevisiae* P27882 Opisthokonta (Fungi) 8.00E-39	*O. tauri* XP_003079772.1 Archaeplastida (Plantae) 1.00E-45
D.discoideum	*M.truncatula* EH25915 Archaeplastida (Plantae) 6.00E-21	*A. mississipp*. XP_006266995 Opisthokonta (Animalia) 4.00E-29	*P.humanus* EEB14183 Opisthokonta (Animalia) 1.00E-03	*Blastocystis* sp. AFL03352 Chromaleolata 0.0		*S.cerevisiae* A0A023ZHL4 Opisthokonta (Fungi) 2.00E-42	*S.salar* ACI69535 Opisthokonta (Animalia) 6.00E-08	*R. microsporus* ORE05664.1 Opisthokonta (Fungi) 1.00E-113	*C.capitata* JAB87019 Opisthokonta (Animalia) 1.00E-39	A: *Ch. cucurbit*. OBZ81755 Archaeplastida (Plantae) 7.00E-39 B: *P.brassicae* (CEO94839.1) Rhizaria 4.00E-24
*D.fasciculatum*	*O.sativa* Q5JN36 Archaeplastida (Plantae) 2.00E-23	*Ch.reinhardtii* EDP02285 Archaeplastida (Plantae) 2.00E-29	*P.hsultus* BAM19942 Opisthokonta (Animalia) 3.00E-02	*T.chinensis* XP_006169972 Opisthokonta (Animalia) 0.0		*L.mirantina* SCV03152.1 Opisthokonta (Fungi) 1.00E-39	*Tetraselmis* sp. JAC74447 Archaeplastida (Plantae) 3.00E-12	*L. oculatus* XP_015207227.1 Opisthokonta (Animalia) 2.00E-121	*C.capitata* JAB87019 Opisthokonta (Animalia) 2.00E-40	A:*A.queenslandica* XP_001782477.1 Opisthokonta (Animalia) 2.00E-39 B: *P. patens* XP_001782477.1 Archaeplastida (Plantae) 3.00E-30
*D.purpureum*	*M.truncatula* KEH25915 Archaeplastida (Plantae) 9.00E-19	*A. mississipp.* XP_006266995 Opisthokonta (Animalia) 1.00E-29	*P.xuthus* BAM19942 Opisthokonta (Animalia) 5.00E-02	*Blstocystis* sp. AFL03352 Chromaleolata 0.0		*T.rangeli* AGN32911 Excavata 6.00E-33	*H.sapiens* NP_653237 Opisthokonta (Animalia) 6.00E-09	*S.formosus* XP_018602154.1 Opisthokonta (Animalia) 2.00E-113	*A.mellifera* XP_001120016 Opisthokonta (Animalia) 1.00E-39	A: *Ch.cucurbit*. OBZ81755.1 Opisthokonta (Fungi) 8.00E-38 B: *S.lenta* WP_028771001.1 Bacteriacea 5.00E-16
*P.pallidum*	*T.cacao* EOY04940 Archaeplastida (Plantae) 3.00E-15	*A.carolinensis* XP_003218148 Opisthokonta (Animalia) 4.00E-28	*S.mansoni *CCD79254 Opisthokonta (Animalia) 4.00E-02	*Blastocystis* sp. AFL03352 Chromaleolata 0.0		*T.suis* KHJ48397 Opisthokonta (Animalia) 4.00E-43	*D.reiro* Q6DE18 Opisthokonta (Animalia) 5.00E-14	*C. anguillulae* ORZ33374.1 Opisthokonta (Fungi) 1.00E-118	*A.mellifera* XP_001120016 Opisthokonta (Animalia) 2.00E-40	A: *M. elongata* OAQ36602.1 Opisthokonta (Fungi) 2.00E-39 B: *S.kowalevskii* XP_006817147.1 Opisthokonta (Animalia) 4.00E-28
*E.dispar/ E.nuttalli*				*Blastocystis sp.* AFL03352 Chromaleolata 0.0						

In the case of the predicted subunits of the PAM complex and OXA complex, the phylogenetic origin of proteins regarded as the best match appeared to be more variable. For the PAM complex subunits the similarity concerned the cognate proteins of Opisthokonta (fungi and animals), Archaeplastida, Chromalveolata and Excavata. In the case of the predicted Pam16, the highest similarity was observed for Opisthokonta (fungi) proteins (*A. castellanii* both isoforms) and Archaeplastida proteins (slime molds). For the predicted Pam18, the highest similarity was observed for Archaeplastida and Opisthokonta (animals) cognate proteins (*A. castellanii* Pam18A and Pam18B, respectively) whereas the slime mold Pam18 predicted proteins displayed the highest similarity to Opisthokonta (animals) cognate proteins with exception of *D. fasciculatum* (Archaeplastida). The predicted Tim44 proteins were the most similar to Opisthokonta (fungi) protein (*A. castellanii*) and Opisthokonta (animal) cognate proteins (all slime molds). Interestingly, all the predicted mtHsp70 proteins displayed the highest similarity to Chromalveolata cognate proteins, with the exception of *D. fasciculatum*, for which the predicted mtHsp70 appeared to be the most similar to an Opisthokonta (animal) protein. The predicted Mge1 appeared to be the most divergent in regard to phylogenetic relationships, as we observed the best match for Opisthokonta (fungi, animal) proteins (slime molds: *D. discoideum, P. pallidum*, and *D. fasciculatum,* respectively), Chromalveolata proteins (*A. castellanii*), and Excavata (*D. purpureum*). The same applies to the slime mold predicted Oxa1B isoform that appeared to be a homolog of the predicted *A. castellanii* Oxa1 (Fig. 3d). The latter was the most similar to an Archaeplastida (plants) cognate protein but the slime mold predicted Oxa1B was the most similar to the cognate proteins of Bacteriaceae, Rhizaria, Archaeplastida (plants), and Opisthokonta (animals), depending on the studied species. However, the second predicted slime mold Oxa1 isoform; i.e. Oxa1A displayed the highest similarity to the Opisthokonta (fungi and animals) and Archaeplastida proteins.

Summing up, the phylogenetic position of the predicted subunits indicated a comparable level of diversity between *A. castellanii* and the slime molds as well as between the slime molds themselves. In the latter, the differences concerning the best match were the most pronounced for *D. fasciculatum.* However, this observation could not be verified by the involvement of the transcriptome dataset into analysis. Moreover, the obtained results revealed a higher level of diversity in the case of the PAM and OXA complexes. Nevertheless, the predicted subunits were mostly similar in their amino acid sequences to the cognate proteins of Opisthokonta (fungi and animals).

## Discussion

The differences in the subunit organization of the mitochondrial protein import complexes have been mainly reported for members of different phylogenetic lineages, while for members of the same clade the issue is poorly studied. Thus, the data are missing to address the evolutionary aspects of the mitochondrial protein import machinery. As mentioned in the Introduction, the Amoebozoa includes taxa of both biomedical and evolutionary importance. We have recently shown comprehensive analyses of the mitochondrial outer membrane import complexes, i.e. the TOM and TOB/SAM complexes, of members of this supergroup, based on searching of the available genome and transcriptome data [14], but the mitochondrial protein import machinery located in the inner membrane and intermembrane have not been addressed.

Accordingly, the PubMed data searching for the Amoebozoa protein import complexes located in these mitochondrial compartments presents only results of a hidden Markov-model-based analysis performed for the *D. discoideum* and *E. histolytica* Tim9-Tim10-Tim12, TIM22, TIM23, and PAM complexes [12, 13, 28]. They predict the presence of the following subunits of the complexes for *D. discoideum*: Tim22 and undefined small Tim proteins (the TIM22 and Tim9-10-12 complexes), Tim17, Tim21, and Tim23 (the TIM23 complex) as well as Pam16, Pam18, and mtHsp70 (the PAM complex), whereas in *E. histolytica*, only mtHsp70 and a possible homolog of Mge1 have been predicted. Moreover, AIF was identified for *D. discoideum* [29]. Thus, we performed the analysis of genome and transcriptome sequences available for different amoebozoans to obtain a more coherent picture. The analysis includes the Tim9-Tim10-Tim12, TIM22, TIM23, PAM, MIA, and OXA complexes (Fig. 2) of organisms representing different subclades and subdivisions of the Amoebozoa (Fig. 1). In general, all the predicted subunits are conserved between *S. cerevisiae* and the studied amoebozoans.

For *E. dispar* and *E. nuttalli* only one subunit of all the studied complexes was predicted, namely mtHsp70, a member of the PAM complex. It is assumed that all *Entamoeba* species have mitosomes, although convincing data are still missing (Graham Clark, personal communication). Similarly, in contrast to the well-known pathogenic *E. histolytica* and *E. invadens*, the pathogenicity of *E. dispar* and *E. nuttalli* is still a matter of debate [15–17]. Nevertheless, it cannot be concluded that the predicted extreme reduction of the protein import apparatus of the mitochondrial inner membrane and the intermembrane space is a marker of a parasitic lifestyle, as parasitic protists are known to express Tim and Pam proteins [4, 13]. On the other hand, for the studied entamoebas the reduction of the protein import complexes in the inner membrane and intermembrane space seems to be much more pronounced than that of the outer membrane ones, where at least channel-forming subunits are predicted to be present [14]. Accordingly, Tom40 and Tob55/Sam50 as well as Mge1 and mtHsp70 were detected for *E. histolytica* [13]. However, for other mitosome possessing organisms the higher numbers of the import complex subunits have been predicted, namely Tom55/Sam50, Tom70, Tim17, Tim21, Tim23, Tim22 for *Encephalitozoon cuniculi* and Tom40, Pam16, Pam18, mtHsp70, Mge1 for *Giardia lamblia* [13]. Thus, the reduction of the protein import complexes appears to be an indicative feature of mitosomes but it seems that the level of the reduction is multifarious. It should also be remembered that mitosomes may contain proteins that are too divergent to be homologs of known import complex subunits, and consequently cannot be found using only bioinformatic tools [30]. For example, the presence of a novel mitosome outer membrane β-barrel protein and other two inner membrane proteins of unknown function has been shown to be unique to *E. histolytica* mitosomes [4, 17]. These proteins do not display similarity to canonical import complex subunits. Moreover, the β-barrel protein named MBOMP30 represents seventh MBOMP subclass lacking any recognizable sequence similarity to any of the six previously identified ones. On the other hand the lack of the transcriptome datasets of *E. dispar* and *E. nuttalli* may impact the possibility of protein prediction.

For *A. castellanii* and the slime molds, the organization of the studied complexes is very similar with exception of the MIA complex (Table 1). Moreover, the predicted organization appears to be quite similar to the canonical one described for *S. cerevisiae* (Fig. 2), Thus it appears that also in these amoebozoan mitochondria the diversity of the inner membrane and the intermembrane space import complexes is less pronounced than in the case of the outer membrane ones [31]. Accordingly, the encoding genes of the slime molds contain similar numbers of predicted exons, being not distinctly different from the numbers predicted for *A. castellanii* putative genes, with the exception of genes encoding Tim50, Mgr2, Tim44, mtHsp70, and Erv1 (Additional file 1: Table S3).

However, a distinct difference is apparent when the predicted subunits of the *A. castellanii* and slime mold Tim9-Tim10-Tim12, PAM, and OXA complexes are compared. Namely, the predicted *A. castellanii* Tim9, Tim10, Pam16, and Pam18 and the slime mold Oxa1 appear to have isoforms encoded by separate genes. Interestingly, searching of available databases indicates the presence of Tim9, Tim10, and Pam protein isoforms in mitochondria of other organisms, mainly animals and humans [7, 9]. Isoforms were also found for human Tim17 [32] and Tim8 [33–35]. In the case of the OXA complex, two genes encoding Oxa1 and Oxa2 have been reported for Opisthokonta (animals and fungi) and more than two for Archaeplastida (plants) [36]. On the other hand, members of the Excavata seem to have isoforms of Oxa2, while Oxa1 is not present, whereas only one gene encoding Oxa protein and a lack of the protein isoforms have been shown for Chromalveolata [7, 36, 37]. Thus the diversity of the OXA complex organization is still to be explained. Accordingly, the precise roles of the paralogs, including Oxa1 and Oxa2, have yet to be clarified [7]. The difference concerning the presence of small Tims, Pam16, Pam18, and Oxa1 isoforms between the studied amoebozoans constitutes an interesting observation from the point of view of the supergroup phylogeny, as the Amoebozoa are regarded as a sister clade to the Opisthokonta [1, 2]. While the putative expression of the *A. castellanii* Tim9, Tim10, Pam16, and Pam18 isoforms resembles Opisthokonta cognate proteins, the possible presence of the slime mold Oxa1 isoforms is much more difficult to explain. Nevertheless, the differences may reflect the evolutionary history of the subclade formation.

As mentioned above, the difference is also observed for the MIA complex as only Erv1 protein is predicted for the *A. castellanii* MIA complex, whereas Erv1, Mia40 and AIF are predicted for all the studied slime molds. Interestingly, as in the case of plant and mammalian but not *S. cerevisiae* mitochondria [38], the predicted slime mold Mia40 appears to be a soluble protein (not shown). Therefore, one can suggest that AIF can serve as Mia40 receptor not only in mammalian mitochondria [39–41]. On the other hand, the apparent absence of Mia40 regarded as an essential protein (e.g. [38]) in some eukaryotes has also been reported. For example, the protein does not seem to be present in brown algae [31] and parasitic protists, such as *Plasmodium falciparum*, *Leishmania tarentolae*, and *Trypanosoma cruzi* [42]. Accordingly, *A. castellanii* is the only proven parasite among the studied amoebozoans, defined as an opportunistic pathogen responsible for amoebic keratitis and granulomatous amoebic encephalitis in humans [43, 44]. Yet, the conclusions in regard to the correlation between the lack of Mia40 and pathogenicity are at present unjustified. Importantly, the presence of small Tim proteins which undergo oxidative folding, indicate that an oxidative folding machinery exists in *A. castellanii* mitochondria [45]. It suggests that other protein(s) can replace the subunit. Accordingly, for the *Trypanosoma brucei* TOM complex, novel receptors with no homology to known TOM import proteins i.e. ATOM46 and ATOM69, have been identified [46]. Interestingly, these proteins were also found in all kinetoplastids (including the non-pathogenic ones), showing that the expression of the unique proteins is not an adaptation to the parasitic lifestyle of *T. brucei* [47].

Interestingly, amino acids sequence comparison (Table 2) indicates that the predicted subunits of the studied complexes display different levels of amino-acid sequence conservation. The lowest diversity is observed for subunits predicted for the TIM23 and MIA complexes whereas the highest diversity occurs for subunits predicted for the OXA complex, particularly in the case of the slime mold Oxa1B isoform that appears to be a homolog of *A. castellanii* Oxa1. The differences are not reflected in the encoding gene intron-exon structures (Additional file 1: Table S3), as it has been observed for the outer membrane complexes that excludes different regulation of protein expression at the level of splicing known to support an adaptation to a given lifestyle [14]. Nevertheless, it can be suggested that the different levels of amino acid sequence variability observed for subunits predicted for the inner membrane and intermembrane space import complexes and the outer membrane import complexes may result from natural selection, as the proteins appear to control efficiently organelle biogenesis and function, including both membranes. Accordingly, as it is speculated that differences in amino acid sequences observed between members of different supergroups reflect the early diversification of eukaryotes [47], it can be proposed that within a given supergroup, the variability may reflect emergence of species within its branches.

## Conclusions

Here we present results of a comprehensive bioinformatic analysis of the protein import complexes located in the mitochondrial inner membrane (the TIM22, TIM23, PAM, and OXA complexes) and intermembrane space (the Tim9-Tim10-Tim12 and MIA complexes) of selected species of the Amoebozoa. This analysis was based on searching of the available genome and transcriptome sequences. Our results indicate that in *A. castellanii* and slime molds, the complex organization appears to be quite similar to the canonical ones described for *S. cerevisiae*. However, distinct differences are observed in amino acid sequences of some of the predicted proteins and in the numbers of some of the protein isoforms. Moreover, the performed analysis for entamoebas, i.e. *E. dispar* and *E. nuttalli*, indicates the absence of any subunit of all the studied complexes except mtHsp70 (the PAM complex), and that further supports the suggestion that all entamoebas have mitosomes. Importantly, the reduction of the protein import apparatus of entamoebas is much more pronounced than in the case of the outer membrane. It seems to be a rule for entamoebas, including *E. histolytica*, but not for all organisms possessing mitosomes. This constitutes an interesting issue from the evolutionary and biomedical perspective, as it addresses the problem of mitochondrial and/or mitosome protein import machinery variability within the currently defined supergroup of eukaryotes. On the other hand, it is becoming clear that the knowledge of mitochondrial protein import of model organisms cannot be generally transferred to all other eukaryotes.

## Additional files

Additional file 1: Table S1. Availability of the genome and transcriptome data of the studied amoebozoans: *Acanthamoeba castellanii (Ac)* [6]*, Dictyostelium discoideum (Dd)* [48], *Dictyostelium fasciculatum (Df)* [49], *Dictyostelium purpureum (Dp)* [50] *Polysphondylium pallidum (Pp)* [48], *Entamoeba nuttalli (En)* [51], and *Entamoeba dispar (Ed)* [52]. **Table S2.** Reference sequences from various eukaryotic lineages for subunits of the small Tims, TIM22, TIM23, PAM, MIA, and OXA complexes obtained by BLAST search. **Table S3.** Numbers of exons in genes encoding the predicted subunits for the studied complexes of selected Amoebozoa: *Acanthamoeba castellanii* (*A.c*)*, Dictyostelium discoideum* (*Dd*)*, D. fasciculatum* (*Df*)*. D. purpureum* (*Dp*)*, Polysphondylium pallidum* (*Pp*)*, Entamoeba dispar* (*Ed*)*,* and *E. nuttalli* (*En*), compared to selected representatives of fungi, animals, and plants. **Figure S1.** Alignment of the predicted subunits of the studied complexes displaying differences in amino acid sequences as compared to their counterparts deposited in the GenBank: (A) *Acanthamoeba castellanii* Tim9A; (B) *Polysphondylium pallidum* Tim9; (C) *A. castellanii* Tim10B; (D) *A. castellanii* Tim22; (E) *A. castellanii* Tim50; (F) *A. castellanii* Mgr2; (G) *A. castellanii* Pam16A; (H) *A. castellanii* Pam16B; and (I) *A. castellanii* mtHsp70. **Figure S2.** *Acanthamoeba castellanii* sequences predicted by transcriptome analysis. Amino acid and mRNA assembly correction for the mitochondrial inner membrane and intermembrane space proteins in *A. castellanii* based on transcriptome and genome assemblies. (DOCX 334 kb)

## Abbreviations

AIF: Apoptosis inducing factor
MIA: Mitochondrial intermembrane space assembly
MIM: Mitochondrial import machinery
OXA: Oxidase assembly factor
PAM: Presequence translocase-associated motor
small Tims: i.e. Tim8-Tim13 and Tim9-Tim10-Tim12 intermembrane space complexes
TIM22: Translocase of the inner membrane 22
TIM23: Translocase of the inner membrane 23
TOB/SAM: Topogenesis of the mitochondrial outer membrane ß-barrel proteins/sorting and assembly machinery
TOM: Translocase of the outer membrane
