# Supplementary material for: The emerging picture of the mitochondrial protein import complexes of Amoebozoa supergroup
Source: BMC Genomics. 2017 Dec 29;18:997. doi: 10.1186/s12864-017-4383-1 (PMC5747110; doi:10.1186/s12864-017-4383-1)
Supplement: Supplementary file 1 — Availability of the genome and transcriptome data of the studied amoebozoans: Acanthamoeba castellanii (Ac) [6], Dictyostelium discoideum (Dd) [48], Dictyostelium fasciculatum (Df) [49], Dictyostelium purpureum (Dp) [50] Polysphondylium pallidum (Pp) [48], Entamoeba nuttalli (En) [51], and Entamoeba dispar (Ed) [52]. Table S2. Reference sequences from various eukaryotic lineages for subunits of the small Tims, TIM22, TIM23, PAM, MIA, and OXA complexes obtained by BLAST search. Table S3. Numbers of exons in genes encoding the predicted subunits for the studied complexes of selected Amoebozoa: Acanthamoeba castellanii (A.c), Dictyostelium discoideum (Dd), D. fasciculatum (Df). D. purpureum (Dp), Polysphondylium pallidum (Pp), Entamoeba dispar (Ed), and E. nuttalli (En), compared to selected representatives of fungi, animals, and plants. Figure S1. Alignment of the predicted subunits of the studied complexes displaying differences in amino acid sequences as compared to their counterparts deposited in the GenBank: (A) Acanthamoeba castellanii Tim9A; (B) Polysphondylium pallidum Tim9; (C) A. castellanii Tim10B; (D) A. castellanii Tim22; (E) A. castellanii Tim50; (F) A. castellanii Mgr2; (G) A. castellanii Pam16A; (H) A. castellanii Pam16B; and (I) A. castellanii mtHsp70. Figure S2. Acanthamoeba castellanii sequences predicted by transcriptome analysis. Amino acid and mRNA assembly correction for the mitochondrial inner membrane and intermembrane space proteins in A. castellanii based on transcriptome and genome assemblies. (DOCX 334 kb) [file 12864_2017_4383_MOESM1_ESM.docx]

**Additional file 1**

**Table S1.** Availability of the genome and transcriptome data of the studied amoebozoans: *Acanthamoeba castellanii* *(Ac)* [6]*, Dictyostelium discoideum (Dd)* [48], *Dictyostelium fasciculatum (Df)* [49], *Dictyostelium purpureum (Dp)* [50] *Polysphondylium pallidum* *(Pp)* [49], *Entamoeba nuttalli (En)* [51], and *Entamoeba dispar (Ed)* [52].

|  | **NCBI Genome Project ID** | **Genome size [Mb]** | **Availability of transcriptome** |
| --- | --- | --- | --- |
| ***Ac*** | PRJNA66753  _AC_RNASeq_  DDBJDRA006231 | 42.02 | +  + |
| ***Dd*** | PRJNA201 | 34.21 | + |
| ***Df*** | PRJNA40189 | 31.02 | - |
| ***Dp*** | PRJNA65531 | 32.96 | + |
| ***Pp*** | PRJNA46447 | 32.97 | + |
| ***Ed*** | PRJNA28615 | 30.63 | - |
| ***En*** | PRJNA257309 | 14.40 | - |

**Table S2.** Reference sequences from various eukaryotic lineages for subunits of the small Tims, TIM22, TIM23, PAM, MIA, and OXA complexes obtained by BLAST search.

| **Protein** | **Supergroup, GenBank accession number and organism** |
| --- | --- |
| **Tim9** | Amoebozoa: Q559H1.1 *D. discoideum* Cryptophyta: CAJ73651.1 *G. theta* Fungi: P0CR97.1 *C. neoformans var. neoformans* B-3501A, Q6BU42.2 *D. hansenii* CBS767, Q4IB65.1 *F. graminearum* PH-1, CCA39785.1 *K. pastoris* CBS 7435, AAN17751.1 *N. crassa*, BAF02263.1 *O. methanolica*, CDO66465.1 *P. reichenowi*, CDM27171.1 *P. roqueforti* FM164, O74700.1 *S. cerevisiae* S 288c, Q6C6Z2.1 *Y. lipolytica* CLIB122 Metazoa: NP_001156116.1 *A. pisum* Q2KIV2.1, *B. taurus* Q61TH2.1, *C. briggsae* ACO15182.1, *C. clemensi*, Q9VYD7.1, *D. melanogaster* ACO07440.1 *O. mykis* Stramenopiles: CBJ27573.1 *E. siliculosus* Viridiplantae: Q9XGX9.2 *A. thaliana*, EDP09353.1 *Ch. reinhardtii*, EXC10652.1 *M. notabilis*, JAC66927.1 *Tetraselmis* *sp*. GSL018 |
| **Tim10** | Amoebozoa: EAL64919.1 *D. discoideum*, AX4 EGG20729.1 *D. fasciculatum*, EFA78336.1 *P. pallidum* PN500 Fungi: Q4WJX5.1 *A*. *fumigatus* Af293, CCU78092.1 *Blu B.meria graminis f. sp. hordei* DH14, CCG23654.1 *C. orthopsilosis*, EGX95429.1 *C. militaris* CM01, EHK96240.1 G. lozoyensis 74030 Q6CIK7.1, *K. lactis* NRRL Y-1140, CCA38002.1 *K. pastoris* CBS 7435, ELQ41567.1 *M. oryzae* Y34, AAK26645.1 *N. crassa*, EEH47798.1 *P. brasiliensis* Pb18, CDM26444.1 *P. roqueforti* FM164, ETS74824.1 *Pestalotiopsis fici* W1061, P87108.1 *S. cerevisiae* S288c, ESZ91432.1 *S. borealis* F-4157 KFX43560.1, *T. marneffei* PM1 EGD89544.1, *T. rubrum* CBS 118892, EEP81759.1 *U. reesii* 1704, XP_009657852.1 *V. dahliae* VdLs.17, EIM22241.1 *W. sebi* CBS 633.66, CCH40739.1 *W. ciferrii*, Q6C6U1.1 *Y. lipolytica* CLIB122 Metazoa: XP_003251707.1 *A. mellifera*, NP_001037412.2 *B. mori*, Q2NKR1.1 *B. taurus*, Q61BP6.1 *C. briggsae*, ACO14987.1 *C. clemensi*, Q6DI06.1 *D. rerio*, NP_611606.1 *D. melanogaster*, AAH32133.1 *H. sapiens*, ADO28878.1 *I. punctatus*, ADD38100.1 *L. salmonis*, Q5RDJ0.1 *P. abelii*, JAA56716.1 *R. pulchellus*, KFM72902.1 *S. mimosarum*, NP_001089442.1 *X. laevis*, KDR10665.1 *Z. nevadensis* Stramenopiles: CBN74778.1 *E. siliculosus* Viridiplantae: NP_001154540.1 *A. thaliana*, EDO96752.1 *Ch*. *reinhardtii*, EMS66511.1 *T. urartu*, AFW67107.1 *Z. mays*, XP_003597417.1 *M. truncatula* |
| **Tim12** | Fungi: CCG23717.1 *C. orthopsilosis*, DAA07212.1 *S. cerevisiae* S288c, BAO42179.1 *K. marxianus* DMKU3-1042, CDH13735.1 *Z. bailii* ISA1307 |
| **Tim18** | Fungi: CAB66444.2 *S. pombe*, EDN63622.1 *S. cerevisiae* YJM789, EEB07311.1 *S. japonicus* yFS275 |
| **Tim22** | Fungi: EFE36371.1 *A benhamiae* CBS 112371, Q75E80.1 *A. gossypii* ATCC 10895, KEY78661.1 *A. fumigatus* var. RP-2014, EEQ47348.1 *C albicans* WO-1, XP_008726323.1 *C. carrionii* CBS 160.54, ADV21055.1 *C. gattii* WM276, Q6BT35.1 *D. hansenii* CBS767, CCT67129.1 *F. fujikuroi* IMI 58289, BAO39189.1 *K. marxianus* DMKU3-1042, CCA37313.1 *K. pastoris* CBS 7435, CDH61513.1 *L. corymbifera* JMRC:FSU:9682, CDI53417.1 *M. pennsylvanicum* 4, XP_007843339.1 *M. roreri* *MCA 2997, XP_007579699.1 N. parvum UCRNP2, AAK26643.1 N. crassa, ESX00038.1 O. parapolymorpha DL-1, CDM29772.1* P. roqueforti FM164, GAK64531.1 *P. antarctica*, KEP51386.1 *R. solani* 123E, CAA98795.1 *S. cerevisiae*, EEB06597.1 *S. japonicus* yFS275, XP_007306139.1 *S. hirsutum* FP-91666 SS1, XP_008043212.1 *T. versicolor* FP-101664 SS1, KDB33441.1 *T. rubrum* D6, EIM19447.1 *W. sebi* *CBS 633.66, CCH42552.1 W. ciferrii, Q6BZY4.2 Y. lipolytica CLIB122, CDH13451.1 Z. bailii ISA1307* Metazoa*: JAC54813.1 B*. dorsalis, KFO07658.1 *B. regulorum* *gibbericeps*, JAC04902.1 *C. capitata*, XP_009556810.1 *C. canorus*, AAW31508.1 *D. melanogaster*, XP_*009632204.1 E. garzetta, ACO12987.1 L. salmonis, KFW86480.1 M. vitellinus, NP_001278090.1 M. musculus, XP_009462838.1 N. nippon, ETE57802.1 O. hannah*, XP_001153337.1 *P. troglodytes*, XP_009489566.1 *P. crispus*, AAF28360.1 *R. norvegicus*, ACI66743.1 *S. salar*, KGB36080.1 S. *haematobium,* XP_009093020.1 *S. canaria*, CEF68097.1 *S. ratti*, XP_009671321.1 *S. camelus australis*, KGL80705.1 *T. guttatus*, NP_001011397.1 X. *(Silurana) tropi*calis Stramenopiles: CBJ27223.1.1 *E. siliculosus* Viridiplantae: EMT23267.1 *A. tauschii*, AEE83706.1 *A. thaliana*, KEH30670.1 *M. truncatula*, EMS56357.1 *T. urartu*, XP_002278395.*1 V. vinifera*, ACG43486.1 *Z. mays* |
| **Sdh3** | Alveolata: AHA41636.1 C. sp. *Vietnam*, AHX80428.1 *H. physophora* Cryptophyta: ACC78218.*1 H. andersenii* Fungi: EKV48833.1 *A.* *bisporus var. bisporus* H97, CCU79401.1 *B.* graminis f. sp. hordei DH14, KDQ32190.1 *P. ostreatus* PC15, EXX77071.*1 R. irregularis* DAOM 197198w, EDN60028.1 *S. cerevisiae* YJM789, NP_587712.1 *S. pombe* 972h- Glaucocystophyceae: AIM52070.1 *C. gloeocystis*, AIU44695.1 *C. paradoxa*, AAK77128.1 *O. triangularis* subsp. *papilionacea* Jakobida: AGH24105.1 *J.* bahamiensis, AGH24238.1 J. libera, YP_0*07890592.1 H. aroides* Metazoa*: AAH20808.1 H. sapiens Rhodophyta: AGO19317.1 G. vagum, AIM52035.1 G. wittrockiana*, AGO19292.1 *G. angusta*, AHG98605.1 *K. striatu*s, ADR03241.1 *P. pulvinata,* AHB34963.1 *P. perforata*, AGL96411.1 *P.* *tene*ra, AGO19267.1 *R. pseudopalmata*, AGU16684.1 *S. durum*, AID57267.1 *W. schizophylla* Stramenopiles: EGB07934.1 A. *anophagefferens,* *CBN78408.1 E. siliculosus, EWM21145.1 N. gaditana Tsukubamonadidae: BAO51989.1 T. globosa* Metazoa: *NP_194948.2* A. thaliana, AIG89919.1 C. annuum, AAP92199.1 Ch. vulgaris, EDP09245.1 Ch. reinhardtii, AEN56102.1 C. melo subsp. melo, ACH42356.1 E. californica, AEB39974.1 F. hygrometrica, AAK73693.1 G. max, AGJ90402.1 L. tulipifera, AGZ90345.1 M. stagnorum, ACH42355.1 N. tetragona, AAK73695.1 O. sativa, AHX80427.1 P. campestris, CDO63299.1 P. reichenowi, AAK77127.1 P. peltatum, AGW52229.1 P. coloniale, AIB08845.1 R. stricta, ADW96025.1 R. communis, AGZ90396.1 R. obtusa, AAK77126.1 S. lycopersicum, JAC78272.1 Tetraselmis sp. GSL018, AGL75423.1 U. gibba, CAQ77586.1 V. vinifera |
| **Tim54** | Fungi: *Q758C9.2 A. gossypii ATCC 10895, KEY82141.1 A. fumigatus var. RP-2014, XP_008597756.1 B. bassiana ARSEF 2860, GAD94582.1 B. spectabilis No. 5, P48990.2 C. albicans SC5314, ENH84263.1 C. orbiculare MAFF 240422, EFI28335.1 C. cinerea okayama7#130, KGB80008.1 C. gattii R265, Q6BTN1.2 D. hansenii CBS767, ESU06476.1 F. graminearum PH-1, EHK99204.1 G. lozoyensis 74030, Q6CLP4.1 K. lactis NRRL Y-1140, EKG11310.1 M. phaseolina MS6, EKD13137.1 M. brunnea f. sp. 'multigermtubi' MB_m1, ESK94180.1 M. roreri MCA 2997, AAK26641.1 N. crassa, ESX02142.1 O. parapolymorpha DL-1, EEH40782.1 Paracoccidioides sp. 'lutzii' Pb01, CDM36832.1 P. roqueforti FM164, EUC66318.1 R. solani AG-3 Rhs1AP, P47045.1 S. cerevisiae S288c, NP_596696.1 S. pombe 972h-, KFX47577.1 T. marneffei PM1, Q6C7Y5.1 Y. lipolytica CLIB122* |
| **Tim29** | Metazoa: NP_612367.1 *H.sapiens*; NP_001018397 *D.reiro;* XP_008055776.1  *C.syrichta; XP_005336210.1 I. tridecemlineatus* |
| **Tim17** | Alveolata*: EDO05838 B. bovis, XP_667663 C. hominis, TU502XP_002142797 C. muris, RN66CDI82752 E. acervulina, CDJ47438 E. bruneti, CDI75559 E. praecox, KEP67510 H. hammondi, EGR32651 I. multifiliis, EJY69210 O. trifallax, GAB67222 P. cynomolgi strain BKF, G44196 T. gondii GAB2-2007-GAL-DOM2, BAM39444 T. orientalis strain Shintoku, XP_001014130 T. thermophila Amoebozoa: ELR15404 A. castellanii str. Neff, Q54K35 D. discoideum Euglenozoa: EPY38671 A. deanei, ADL27531 L. donovani, CAM65941 L. infantum JPCM5, EPY34375 S. culicis, XP_829386 T. brucei brucei TREU927 Fungi EEH05746 A. capsulatus, G186AR EGE77577 A. dermatitidis ATCC 18188, EDP54083 A. fumigatus A1163, XP_659496 A. nidulans FGSC A4, EIT73455 A. oryzae 3.042, EEQ32139 A. otae CBS 113480, EJP66697 B. bassiana ARSEF 2860, EIF46720 B. bruxellensis AWRI1499, CCU82620 B. graminis f. sp. Hordei DH14, EEQ45884 C. albicans WO-1, EEQ38478 C. lusitaniae ATCC 42720, EGX90965 C. militaris CM01, AAW45378 C. neoformans var. neoformans JEC21, CCG22231 C. orthopsilosis, XP_003069734 C. posadasii C735 delta SOWgp, EER34753 C. tropicalis MYA-3404, XP_386591 F. graminearum PH-1, BAP69527 K. marxianus, BAO37975 K. marxianus DMKU3-1042, CCA37804 K. pastoris CBS 7435, EDK43772 L. elongisporus NRRL YB-4239, XP_003839657 L. maculans JN3, EFY93679 M. acridum CQMa 102, KGG51434 M. sp. UGP3, EAA30931 N. crassa OR74A, ESW97468 O. parapolymorpha DL-1, GAC73962 P. antarctica T-34, EKV09420 P. digitatum Pd1, KEP48336 R. solani 123E, EMS24268 R. toruloides NP11, ESZ91393 S. borealis F-4157, CAA89438 S. cerevisiae, EPY50963 S. cryophilus OY26, XP_001384934 S. stipitis CBS 6054, EEA19313 T. marneffei ATCC 18224, XP_003235933 T. rubrum CBS 118892, EEP81326 U. reesii 1704, KDB14355 V. virens, CCH44686 W. ciferrii, EIM21661 W. sebi CBS 633.66, CDH09464 Z. bailii ISA1307 Haptophyceae: EOD05810 E. huxleyi CCMP1516* Metazoa*: XP_001660550 A. aegypti, JAC08771 A. albopictus, JAC20445 A. cajennense, XP_006613413 A. dorasta, XP_003699051 A. florea, XP_003249498 A. mellifera, ERG81021 A. suum, XP_001899899 B. malayi, JAB59136 C. appendiculata, O44477 C. elegans, AAF53464 D. melanogaster, AAH16817 H. sapiens, EEC09284 I. scapularis, ABM55650 M. hirsutus, ETN68418 N. americanus, JAA94104 P. albipes, BAA21818 R. norvegicus, CCD79606 S. mansoni, JAC18078 T. infestans, CDW53104 T. trichiura, NP_001011183 X. (Silurana) tropicalis* Parabasalia*: EAY21397 T. vaginalis G3 Rhodophyta: BAM83018 C. merolae Stramenopiles: CBK24655 B. hominis, CBN74293 E. siliculosus, EEY55736 P. infestans T30-4 Trimastix: AGH33871, T. pyriformis Viridiplantae: EMT27074 A. tauschii, AAF03749 A. thaliana, EDP07835 Ch. reinhardtii, EEH59917 M. pusilla CCMP1545, ACO68335 M. sp. RCC299, ABF96614 O. sativa Japonica Group, ABO94080 O. lucimarinus CCE9901, AGV54593 P. vulgaris, ERP59569 P. trichocarpa, JAC71760 Tetraselmis sp. GSL018, DAA41392 Z. mays, EMS67233 T. urartu* |
| **Tim21** | Amoebozoa *: ELR18817 A. castellanii str. Neff, EAL61476 D. discoideum AX4* Fungi: *EEH03090 A. capsulatus G186AR, Q75CX4 A. gossypii ATCC 10895, KEQ88358 A. pullulans EXF-150, CCU76993 B. graminis f. sp. hordei DH14, KGQ08271 B. bassiana D1-5, KGT72424 C. albicans 12C, ELA31204 C. gloeosporioides Nara gc5, EAS29397 C. immitis RS, Q6BVK1 D. hansenii CBS767, CCT64030 F. fujikuroi IMI 58289, BAO40849 K. marxianus DMKU3-1042, CCA39860 K. pastoris CBS 7435, CDI52500 M. pennsylvanicum 4, EXV01724 M. robertsii, Q7S8S5 N. crassa OR74A, EKV09414 P. digitatum Pd1, EUC67098 R. solani AG-3 Rhs1AP, EMS22022 R. toruloides, NP11 P53220 S. cerevisiae S288c, EPY50153 S. cryophilus OY26, KFX51919 T. marneffei PM1, XP_003236101 T. rubrum CBS 118892, Q6CAQ9 Y. lipolytica CLIB122, CDH08847 Z. bailii ISA1307* Metazoa*: KFP79180 A. chloris, XP_007254823 A. mexicanus, JAC53599 B. dorsalis, KFO84967 B. rhinoceros silvestris, XP_002757376 C. jacchus, XP_007886198 C. milii, XP_688866 D. rerio, XP_004466859 D. novemcinctus, JAA45898 D. rotundus, CDI96968 E. multilocularis, XP_004703196 E. telfairi, XP_008590831 G. variegatus, XP_419102 G. gallus, XP_004059592 G. gorilla gorilla, KFP94068 H. albicilla, XP_004889998 H. glaber, NP_054896 H. sapiens, XP_005334441 I. tridecemlineatus, JAB80475 I. ricinus, XP_004654873 J. jaculus, XP_006742707 L. weddellii, KFQ02623 L. discolor, XP_007449735 L. vexillifer, XP_005586507 M. fascicularis, XP_008848886 N. galili, XP_003424856 N. vitripennis, XP_512174 P. troglodytes, XP_003914530 P. anubis, NP_001008344 R. norvegicus, XP_010355003 R. roxellana, XP_003353490 S. scrofa, XP_002195546 T. guttata, XP_010222906 T. guttatus, XP_009967481 T. alba, XP_008684115 U. maritimus, XP_006213272 V. pacos, NP_001088133 X. laevis, XP_005483029 Z. albicollis Stramenopiles: CBK24881 B. hominis, CBN78209 E. siliculosus Viridiplantae: Q1G3L1 A. thaliana, KFM24274 A. protothecoides, XP_003569239 B. distachyon, XP_009116149 B. rapa, XP_008456633 C. melo, XP_010052983 E. grandis, XP_008356247 M. domestica, KEH42872 M. truncatula, XP_009391514 M. acuminata subsp. malaccensis, XP_010259181 N. nucifera, XP_009789351 N. sylvestris, CAL52705 O. tauri, XP_008797262 P. dactylifera, XP_008235556 P. mume, XP_009370840 P. bretschneideri, EEF50285 R. communis, XP_004245573 S. lycopersicum, JAC69724 T. sp. GSL018, EOX96407 T. cacao* |
| **Tim23** | Amoebozoa *: XP_004336406.1 A. castellanii* Fung*i: XP_001399276.2 A. niger CBS 513.88, KGQ04518.1 B. bassiana D1-5, EEQ47485.1 C. albicans WO-1, EAU90199.1 C. cinerea okayama7#130, EGX89106.1 C. militaris CM01, BAO41761.1 K. marxianus DMKU3-1042, CCA37158.1 K. pastoris CBS 7435, CBX90712.1 L. maculans JN3, EHA54104.1 M. oryzae 70-15, ESW99619.1 O. parapolymorpha DL-1, BAK62332.1 P. troglodytes, EEH35696.1 Paracoccidioides sp. 'lutzii' Pb01, CDM34340.1 P. roqueforti FM164, GAC77548.1 P. antarctica T-34, CCX30794.1 P. omphalodes CBS 100304, CCO28414.1 R. solani AG-1 IB, AAK26640.1 N. crassa, CAA96296.1 S. cerevisiae, ABN66190.1 S. stipitis CBS 6054, EEB09044.1 S. japonicus yFS275, ESZ93538.1 S. borealis F-4157, EMF10345.1 S. musiva SO2202, KFX50523.1 T. marneffei PM1, CCH41947.1 W. ciferrii, CDH11000.1 Z. bailii ISA1307* Metazoa*: KFP83765.1 A. chloris, EGI68291.1 A. echinatior, EMT08039.1 A. tauschii, EOA96539.1 A. platyrhynchos, JAB67651.1 A. glabripennis, AEY60618.1 A. cerana, KFM00604.1 A. forsteri, ADY44725.1 A. suum, JAC54107.1 B. dorsalis, NP_001077127.1 B. taurus, KFP06749.1 C. anna, EDS34892.1 C. quinquefasciatus, NP_001099068.1 D. rerio, KFQ07887.1 H. albicilla, EHB06681.1 H. glaber, AHH37985.1 I. punctatus, AAI32020.1 M. musculus, EPQ18233.1 M. brandtii, KFZ57885.1 P. cristatus, ELK08353.1 P. alecto, KFM59780.1 S. mimosarum, CEF63828.1 S. ratti, KFV74132.1 S. camelus australis, KFV19655.1 T. erythrolophus, NP_001085062.1 X. laevis, KDR18289.1 Z. nevadensis Stramenopiles: CBK22458.2 B. hominis, CBJ48753.1 E. siliculosus, EWM25356.1 N. gaditana Viridiplantae: AAK31587.1 A. thaliana, KFM28135.1 A. protothecoides, EUB62644.1 E. granulosus, XP_003627207.1 M. truncatula, ABF70068.1 M. acuminate, JAC61579.1 Tetraselmis sp. GSL018, KDB17855.1 V. virens* |
| **b14.7** | Metazoa: CAD52867.1 *B.taurus*, NP_611538.1 *D.melanogaster*; Vidiplantae XP_001689829.1 *Ch. reinhardtii* |
| **Tim50** | *Alveolata: CDO63721.1 P. reichenowi, CDR10761.1 P. chabaudi chabaudi, CDS44516.1 P. berghei ANKA, CDU16138.1 P. yoelii* Fungi*: EAW11412.1 A. clavatus NRRL 1, KGQ12409.1 B. bassiana D1-5, CCU82776.1 B. graminis f. sp. hordei DH14, GAD93398.1 B. spectabilis No. 5, KGU31521.1 C. albicans P34048, EAS33711.2 C. immitis RS, KGB75546.1 C. gattii R265, ENH69514.1 F. oxysporum f. sp. cubense race 1, EHL02335.1 G. lozoyensis 74030, BAP69924.1 K. marxianus, CCA37785.1 K. pastoris CBS 7435, ESK90564.1 M. roreri MCA 2997, EAW24009.1 N. fischeri NRRL 181, AAO32939.1 N. crassa, ESW97253.1 O. parapolymorpha DL-1, EKV07779.1 P. digitatum Pd1, ETS84588.1 P. fici W106-1, CCO33558.1 R. solani AG-1 IB, CEI90874.1 R. microsporus, EGU13482.1 R. glutinis ATCC 204091, DAA11368.1 S. cerevisiae S288c, CAA17836.2 S. pombe, EEA20875.1 T. marneffei ATCC 18224, CEJ94291.1 T. hemipterigena, EGD98204.1 T. tonsurans CBS 112818, EEP76886.1 U. reesii 1704, EEY23820.1 V. alfalfae VaMs.102, CCH44395.1 W. ciferrii, CDH14153.1 Z. bailii ISA1307* Metazoa*: XP_006277800.1 A. mississippiensis, XP_003228426.1 A. carolinensis, ADY41519.1 A. suum, XP_007180037.1 B. acutorostrata scammoni, XP_005890618.1 B. mutus, XP_006068712.1 B. bubalis, EPY85982.1 C. ferus, XP_007618267.1 C. griseus, XP_008307767.1 C. semilaevis, NP_956959.1 D. rerio, XP_004481509.1 D. novemcinctus, EUB60343.1 E. granulosus, XP_010892157.1 E. lucius, XP_003997856.1 F. catus, XP_001233751.3 G. gallus, XP_004060765.1 G. gorilla gorilla, XP_004894730.1 H. glaber, AAT01208.1 H. sapiens, XP_005336476.1 I. tridecemlineatus, JAA66637.1 I. ricinus, XP_004670472.1 J. jaculus, EFO23567.1 L. loa, AFE70158.1 M. mulatta, NP_079892.1 M. musculus, XP_004776326.1 M. putorius furo, XP_006772020.1 M. davidii, XP_004015736.1 O. aries, XP_001138592.1 P. troglodytes, XP_007097054.1 P. tigris altaica, XP_009230856.1 P. abelii, XP_001073346.3 R. norvegicus, CAX82564.1 S. japonicum, XP_003355956.2 S. scrofa, XP_006140443.1 T. chinensis, XP_008686982.1 U. maritimus, XP_006215092.1 V. pacos Rhodophyta: BAM81284.1 C. merolae strain 10D, EME28918.1 G. sulphuraria Stramenopiles: CCA24466.1 A. laibachii Nc14, CBK23956.2 B. hominis, EWM27179.1 N. gaditana, EEY55063.1 P. infestans T30-4 Viridiplantae: AEE33317.1 A. thaliana, KFM28345.1 A. protothecoides, XP_010501219.1 C. sativa, XP_008462988.1 C. melo, XP_010907569.1 E. guineensis, XP_010061466.1 E. grandis, KHN43889.1 G. soja, KHG22376.1 G. arboreum, XP_008385781.1 M. domestica, AET00752.1 M. truncatula, EXB36079.1 M. notabilis, ABO95340.1 O. lucimarinus CCE9901, CEF97279.1 O. tauri, XP_008780990.1 P. dactylifera, XP_011038776.1 P. euphratica, XP_008244589.1 P. mume, XP_011095527.1 S. indicum, XP_010324430.1 S. lycopersicum, JAC70392.1 Tetraselmis sp. GSL018, EMS45392.1 T. urartu, XP_002264515.1 V. vinifera, ACG39671.1 Z. mays* |
| **Mgr2** | *Amoebozoa: EAL64383.1 D. discoideum AX4, EGG15517.1 D. fasciculatum, EFA75083.1 P. pallidum PN500 Fungi: EAW11540.1 A. clavatus NRRL 1, EJP65278.1 B. bassiana ARSEF 2860, EEQ69612.1 B. dermatitidis SLH14081, GAD92126.1 B. spectabilis No. 5, KGQ98449.1 C. albicans P37005, AFR94312.1 C. neoformans var. grubii H99, CCT64575.1 F. fujikuroi IMI 58289, EGC42369.1 H. capsulatum H88, BAO38420.1 K. marxianus DMKU3-1042, CCA39127.1 K. pastoris CBS 7435, CBX94454.1 L. maculans JN3, EFZ03979.1 M. robertsii ARSEF 23, XP_007851737.1 M. roreri MCA 2997, EAA34843.2 N. crassa OR74A, CDM34011.1 P. roqueforti FM164, XP_007922307.1 P. fijiensis CIRAD86, CEI99349.1 R. microsporus, DAA11334.1 S. cerevisiae S288c, NP_595661.1 S. pombe 972h-, KIH93707.1 S. brasiliensis 5110, EED20192.1 T. stipitatus ATCC 10500, CCG81328.1 T. deformans PYCC 5710, XP_007913615.1 T. minima UCRPA7, CEJ90856.1 T. hemipterigena, ETS00496.1 T. reesei RUT C-30, EOQ98895.1 W. ichthyophaga EXF-994, CDH12368.1 Z. bailii ISA1307* Metazoa*: KFP69474.1 A. chloris, EAT39849.1 A. aegypti, XP_006269206.1 A. mississippiensis, AEY57605.1 A. cerana, XP_004929285.1 B. mori, CDP92247.1 B. malayi, XP_006071976.1 B. bubalis, EFO85938.1 C. remanei, ACO14705.1 C. clemensi, AFP07344.1 C. milii, EPY78288.1 C. ferus, XP_005514701.1 C. livia, XP_003500611.1 C. cetulus griseus, XP_001353165.1 D. pseudoobscura pseudoobscura, CDI99120.1 E. multilocularis, XP_004701361.1 E. telfairi, XP_006889272.1 E. edwardii, NP_001185750.1 G. gallus, KFV40653.1 G. stellata, NP_036374.1 H. sapiens, CDS30940.1 H. microstoma, EFO21660.1 L. loa, EHH19738.1 M. mulatta, EDL01934.1 M. musculus, XP_010771935.1 N. coriiceps, XP_003800338.1 O. garnettii, AGU01741.1 P. tigris altaica XP_007439560.1 P. bivittatus, XP_002735645.2 S. kowalevskii, ACI66025.1 S. salar, ABK55622.1 S. scrofa, XP_004327035.1 T. truncates, XP_008689030.1 U. maritimus, NP_001165118.1 X. laevis, XP_005810879.1 X. maculatus XP_005495566.1 Z. albicollis, KDR18366.1 Z. nevadensis* |
| **Pam16** | Alveolata*: EJY77382 O. trifallax, Q4YQD6 P. berghei, CDW86138 S. lemnae, EAS05961.3 T. thermophila SB210* Amoebozoa*: L8GKS8 A. castellanii, EAL66303 D. discoideum AX4, EFA78034 P. pallidum PN500* Fungi*: EAW11829 A. clavatus NRRL 1, G2XZF9 B. fuckeliana, N1JMM1 B. graminis, CCG24763 C. orthopsilosis, EFX00583 G. clavigera kw1407, F2QLR5 K. pastoris, EKG20179 M. phaseolina MS6, EAW19441 N. fischeri NRRL 181, EPE07207 O. piceae UAMH 11346, EQK99091 O. sinensis CO18, EKV05575 P. digitatum Pd1, EUC64253 R. solani AG-3 Rhs1AP, ESZ92181 S. borealis F-4157, EWG90309 S. cerevisiae P301, EMF11060 S. musiva SO2202, EEA22894 T. marneffei ATCC 18224* Metazoa*: Q1HQE3 A. aegypti PE, NP_001156220 A. pisum, XP_010838794 B. bison bison, XP_005901385 B. mutus, NP_001003391 C. lupus familiaris, XP_007891812 C. milii, KFU85304 C. pelagica, XP_005306225 C. picta bellii, XP_005007524 C. porcellus XP_004518222 C. capitata XP_008472978 D. citri XP_004452233 D. novemcinctus NP_957098 D. rerio, XP_010887735 E. Lucius, XP_006942450 F. catus, NP_001004377 G. gallus, XP_004057153 G. gorilla gorilla, XP_008572051 G. variegatus, EFN83007 H. saltator, EAW85314 H. sapiens, XP_009951651 L. discolor, EFO19242 L. loa, AFP62531 M. domestica, XP_010717660 M. gallopavo, EHH31385 M. mulatta, XP_008844521 N. galili, XP_007953788 O. afer afer, EEB15272 P. humanus corporis, XP_007073416 P. tigris altaica, EDM06283 R. norvegicus, XP_010352228 R. roxellana, XP_005662234 S. scrofa, XP_009967857 T. alba, XP_010220480 T. guttatus, XP_004325048 T. truncates, XP_008069318 T. syrichta, EJW80447 W. bancrofti, NP_001004771 X. (Silurana) tropicalis, XP_005497850 Z. albicollis, KDR06593 Z. nevadensis Stramenopiles: CBK24038 B. hominis, D7FSN0 E. siliculosus GN Viridiplantae: AEE79899 A. thaliana, EDP00908 C. reinhardtii, Q5JN36 O. sativa subsp, Q010B6 O. tauri, JAC77490 T. sp. GSL018, EOY22657 T. cacao* |
| **Pam18** | Alveolata*: CDO63701.1 P. reichenowi;* Amoebozoa: *Q54QN1.1 D. discoideum, EGG24887.1 D. fasciculatum, EFA86354.1 P. pallidum PN500;*Fungi*: CBF77258.1 A. nidulans FGSC A4, EAK93469.1 C. albicans SC5314, EOB11887.1 N. bombycis CQ1, CDM33488.1 P. roqueforti FM164, CCO29613.1 R. solani AG-1 IB, EDN59554.1 S. cerevisiae YJM789, ELQ74981.1 T. hominis;* Metazoa: *XP_006266995.1 A. mississippiensis, DAA33298.1 B. taurus, XP_010134502.1 B. rhinoceros silvestris, XP_002758138.1 C. jacchus, XP_007905955.1 C. milii, JAG45036.1 C. horridus, AAI50462.1 D. rerio, XP_004477689.1 D. novemcinctus, XP_004708677.1 E. telfairi, XP_001496054.1 E. caballus, XP_005232995.1 F. peregrinus, XP_422774.1 G. gallus, XP_009818276.1 G. stellata, XP_005419065.1 G. fortis, XP_010571235.1 H. leucocephalus, XP_004907290.1 H. glaber, EAW78363.1 H. sapiens, XP_005330315.1 I. tridecemlineatus, XP_004652370.1 J. jaculus, AFI35270.1 M. mulatta, XP_007502047.1 M. domestica, NP_001273902.1 M. musculus, XP_006084936.1 M. lucifugus, XP_010010823.1 N. notabilis, XP_003256568.1 N. leucogenys, XP_010795482.1 N. coriiceps, XP_001506509.2 O. anatinus, XP_010073138.1 P. gutturalis, XP_006925818.1 P. alecto, XP_007423643.1 P. bivittatus, NP_001128112.1 R. norvegicus, XP_009087310.1 S. canaria, XP_009682167.1 S. camelus australis, NP_001177133.1 S. scrofa, XP_004186245.1 T. guttata, XP_010217242.1 T. guttatus, XP_008703508.1 U. maritimus, NP_001091424.1 X. laevis, XP_005484995.1 Z. albicollis Stramenopiles: CBK21976.1 B. hominis;Viridiplantae: EDP02285.1 Ch. reinhardtii, KHG13401.1 G. arboreum, JAC77459.1 Tetraselmis sp. GSL018* |
| **Tim44** | Alveolata*: CDR94253 B. bigemina, EDO07177 B. bovis, EKX74094 B. equi strain WA, CDJ40014 E. tenella, KEP67693 H. hammondi, CDU18021 P. yoelii,* ESS32755 T. gondii VEG ;Amoebozoa ELR22060 *A. castellanii* str. Neff, EAL64247 *D. discoideum* AX4, EGG20389 D. fasciculatum ;Fungi*: EEH08008 A. capsulatus G186AR, EAW07849 A. clavatus NRRL 1, EJD47973 A. delicata TFB-10046 SS5, EEQ90411 A. dermatitidis ER-3, EED50174 A. flavus NRRL3357, EDP56221 A. fumigatus A1163, GAA89581 A. kawachii IFO 4308, KEQ64573 A. melanogenum CBS 110374, EEQ33398 A. otae CBS 113480, EJP62852 B. bassiana ARSEF 2860, EGE80784 B. dermatitidis ATCC 18188, CCU82961 B. graminis f. sp. hordei DH14, GAD96631 B. spectabilis No. 5, CCG23585 C. orthopsilosis, KHJ33693 E. necator, EJD07340 F. mediterranea MF3/22, EMT70959 F. oxysporum f. sp. cubense race 4, EJT69222 G. graminis var. tritici R3-111a-1, EHL01492 G. lozoyensis 74030, EPQ56376 G. trabeum ATCC 11539, EER39916 H. capsulatum H143, BAO41134 K. marxianus DMKU3-1042, CCA39624 K. pastoris CBS 7435, EFY85667 M. acridum CQMa 102, ELQ33298 M. oryzae Y34, ESK97850 M. roreri MCA 2997, KGG50821 M. sp. UGP3, EAA36093 N. crassa OR74A, EAW22885 N. fischeri NRRL 181, ESW97011 O. parapolymorpha DL-1, EPE08902 O. piceae UAMH 11346, EQL02656 O. sinensis CO18, CDP22532 P. anserina S mat+, GAC77651 P. antarctica T-34, CCX10903 P. omphalodes CBS 100304, CDM30253 P. roqueforti FM164, EEH40848 P. sp. 'lutzii' Pb01, EIN06037 P. strigosozonata HHB-11173 SS5, ELU43659 R. solani AG-1 IA, EMS24410 R. toruloides NP11, KEZ46803 S. apiospermum ,ESZ94737 S. borealis F-4157, KIH89607 S. brasiliensis 5110, Q01852 S. cerevisiae S288c, EPY49522 S. cryophilus OY26, EIM86657 S. hirsutum FP-91666 SS1, CEJ83072 T. hemipterigena EOO02458, T. minima UCRPA7, EED24036 T. stipitatus ATCC 10500, EGD99407 T. tonsurans CBS 112818, EIW58681 T.versicolor FP-101664 SS1, EEP78611 U. reesii 1704, KDB10991 U. virens, EGY22019 V. dahliae VdLs7, CCH42212 W. ciferrii, EIM24066 W. sebi CBS 633.66, CDH14375 Z. bailii ISA1307, EGP91982 Z. tritici IPO323* Metazoa*: XP_001662961 A. aegypti, JAC12055 A. albopictus, KFP90877 A. vittatum, JAC98477 B. cucurbitae, NP_001179583 B. taurus, JAC05589 C. capitata, KFZ46992 C. carolinensis, KFP60946 C. cristata, EFN68959 C. floridanus, EKC17235 C. gigas, EGW03397 C. griseus, JAB02808 C. jacchus, EMC88881 C. livia, KFP35491 C. macqueenii, AFP04356 C. milii, KFP34297 C. striatus, KGL92850 C. vociferous, EHJ63121 D. plexippus, NP_001013525 D. rerio, KFP14207 E. garzetta, EUB62109 E. granulosus, KFW07703 E. helias, KFV93216 F. glacialis, NP_001273092 G. gallus, ADD19374 G. morsitans morsitans, KFQ03542 H. albicilla, EHB04240 H. glaber, EFN80103 H. saltator, O43615 H. sapiens, AHH37835 I. punctatus, JAA71149 I. ricinus, KFQ01376 L. discolor, JAF98439 L. hesperus, ELK38613 M. davidii, AFE70635 M. mulatta, NP_035722 M. musculus, KFR04846 N. nippon, KFQ46741 N. notabilis, ETE57409 O. hannah, KFR11906 O. hoazin, KFW61209 P. adeliae, ELK08081 P. alecto, KFW93625 P. carbo, EEB14183 P. humanus corporis, KFQ65918 P. lepturus, BAK62162 P. troglodytes, BAM19942 P. xuthus, BAA21820 R. norvegicus, JAA61640 R. pulchellus, KFV73981 S. camelus australis, CEF65844 S. ratti, KFV49718 T. alba, KFV03406 T. erythrolophus, KGL82316 T. guttatus, KDR14720 Z. nevadensis Rhodophyta: BAM80316 C. merolae;Stramenopiles: CBJ27872 E. Siliculosus Viridiplantae: EMT23052 A. tauschii, ABE65836 A. thaliana, KFM27788 A. protothecoides, KHN24793 G. soja, KHG20040 G. arboretum, XP_003596691 M. truncatula, EXC05426 M. notabilis, ABO98743 O. lucimarinus CCE9901, EEE80050 P. trichocarpa, EEF37816 R. communis, AEL98685 S. latifolia, JAC68881 T. sp. GSL018, EMS55855 T. urartu* |
| **mtHsp70** | Alveolata : XP_002367417.1 *T. gondii* ME49; Amoebozoa: Q9GU25 *E. histolytica*; Euglenozoa: ACC68074.1 *L. chagasi*; Fungi: CKEQ88014.1 *A. pullulans* EXF-150, CO31741.1 *R. solani* AG-1 IB, CCH43302.1 *W. ciferri*i; Metazoa: EFB18547.1 *A. melanoleuca*, XP_006015787.1 *A. sinensis*, XP_003215333.1 *A. carolinensis*, XP_007172291.1 *B. acutorostrata scammoni*, AAI02335.1 *B. taurus*, AHC29045.1 *B. bubalis*, XP_002744251.1 *C. calithrix jacchus*, XP_006180391.1 *C. ferus*, XP_003477450.1 *C. porcellus*, XP_004420186.1 *C. simum simum*, XP_008634993.1 *C. brachyrhynchos*, AAB62091.1 *C. griseus*, XP_004467076.1 *D. novemcinctus*, NP_523741.2 *D. melanogaster*, XP_006903147.1 *E. edwardii*, XP_008139972.1 *E. fuscus*, NP_001157356.1 *E. caballus*, XP_007517614.1 *E. europaeus*, XP_003980861.1 *F. catus*, NP_001006147.1 *G. gallus*, XP_004042637.1 *G. gorilla gorilla*, XP_004841875.1 *H. glaber*, BAH12688.; *H. sapie*ns, XP_005327316.1 *I. tridecemlineatus*, XP_006739919.1 *L. weddellii*, XP_007461485.1 *L. vexillifer*, XP_003404573.1 *L. africana*, ABK27326.1 *L. variegatus*, XP_001113234.1 *M. mulatta*, NP_034611.2 *M*. *musculus,* XP_006086590.1 *M. lucifugus*, XP_003266489.1 *N. leucogenys*, XP_004008889.1 *O. aries*, XP_003829266.1 *P. paniscus*, JAA02220.1 P*. troglodytes*, NP_001094128.2 *R. norvegicus*, Q6ZZX8 *S. salar*, XP_005661752.1 *S. scrofa*, XP_008054623.1 *T. syrichta*, CDW60853.1 *T. trichiura*, XP_006169972.1 *T. chinensis*, XP_004312108.1 *T. truncatus*, XP_006204644.1 *V. pacos*, NP_001079627.1 *X. laevis*, XP_005493182.1 *Z. albicollis*. |
| **Tim15** | Virdiplatae: XP_003083615.1 *O.tauri*; Fungy: DAA10251.1 *S.cerevisiae* S288C, XP_002175448.1 *S. japonicus* yFS275 Streptophyta: KVH88722.1 *Cynara cardunculus var. scolymus* |
| **Mge1** | Alveolata: XP_001610748 *B. bovis* T2Bo, XP_667827 *C. hominis* TU502, KEP62151 *H. hammondi*, CBZ50929 *N. caninum* *Liverpool*, ESS33648 *T. gondii*; Amoebozoa: EAL65496 *D. discoideum* AX4;Cryptophyta: EKX47499 *G. theta* CCMP2712; Diplomonadida: ESU42581 *G. Intestinalis*;Euglenozoa: EPY43085 *A. deanei*, CAM40232 *L. braziliensis* MHOM/BR/75/M2904, EPY26735 *S. culici,s* XP_803261 *T. cruzi* strain CL Brener, KEG13043 *T. grayi*; Fungi: KEY75448.1 *A. fumigatus* var. RP-2014, EJP68008.1 *B. bassiana* ARSEF 2860, EEQ70541.1 *B. dermatitidis* SLH14081, KHC30774.1 *C. albicans* Ca6, EXF85605.1 *C. fioriniae* PJ7, EAU88536.1 *C. cinerea* *okayama*, EGX96208.1 *C. militaris* CM01, EJU00817.1 *Dacryopinax sp*. DJM-731 SS1, CCT62624.1 *F. fujikuroi* IMI 58289, ETW85921.1 *H. irregulare* TC 32-1, EEH11500.1 *H. capsulatum* G186AR, CAY68106.1 *K. pastoris* GS115, CBX93218.1 *L. maculans* JN3, EFY86707.1 *M. acridum CQMa* 102, EFQ99251.1 *M. gypseum* CBS 118893, ESW98834.1 *O. parapolymorpha* DL-1, EEH35208.1 *Paracoccidioides* *sp. 'lutzii'* Pb01, KGO36484.1 *P. expansum*, GAC73108.1 *P. antarctica* T-34, EUC57853.1 *R. solani* AG-3 Rhs1AP, EMS22669.1 *R*. *toruloides* NP11, EPX71250.1 *S. octosporus* yFS286, KFX48866.1 *T. marneffei* PM1, ETS06423.1 *T. reesei* RUT C-30, EEY19303.1 *V*. *alfalfae* VaMs.102, EIM19083.1 *W. sebi* CBS 633.66;Haptophyceae: EOD28413 *E. huxleyi* CCMP1516;Metazoa: AEY60378.1 *A*. *cerana,* ETN60553.1 *A. darling*, EGI62013.1 *A. echinatior*, BAB18515.1 *A. gossypii*, ADY49350.1 *A. suum*, XP_001892279.1 *B. malayi*, ELR56671.1 *B. mutus*, EPY88691.1 *C. ferus*, EFN66100.1 *C. floridanus*, JAB07354.1 *C. jacchus*, EMP38887.1 *Ch. mydas*, NP_001275592.1 *D. rerio*, NP_001006458.1 *G. gallus*, EHB16017.1 *H. glaber*, NP_689620.2 *H. sapiens*, JAG09385.1 *L. hesperus*, EPQ20402.1 *M. brandtii*, AFP59200.1 *M. domestica*, AFE66971.1 *M. mulatta*, NP_067271.1 *M. musculus*, ETN83853.1 *N*. *americanus*, NP_001127196.1 *P. abelii*, ELK06528.1 *P. alecto*, ACN10134.1 *S. salar*, ELW64311.1 *T. chinensis*; Rhodophyta: CDF39463.1 *Ch. crispus*, BAM83364.1 *C. merolae strain* 10D, EME28433.1 *G. sulphuraria*; Stramenopiles: AFL03360.1 B. sp. NandII CBN76646.1, *E. siliculosus* EWM28579.1, *N. gaditana* EEC48094.1, *P. tricornutum* CCAP 1055/1, EQC32128.1 *S. diclina* VS20, AFA52569.1 *V. litorea*; Viridiplantae: EMT30761.1 *A. tauschii* EFH70175.1 *A. lyrata subsp*. lyrata CCO20645.1 *B. prasinos* *EDP03891.1 Ch. reinhardtii KHN42170.1 G. soja KDD76219.1 Helicosporidium sp. ATCC 50920 ADU56191.1 J. curcas* KEH33928.1 M. truncatula AAC72386.1 *N. tabacum* CEF97491.1 *O. tauri* JAC68908.1 T. sp. GSL018 EOY10409.1 *T. cacao* EMS64517.1 *T. urartu* AFW61923.1 *Z. mays* |
| **Mia40** | Fungi: EFE35822 *A. benhamiae* CBS 112371, EAW11952 *A. clavatus* NRRL 1, XP_001273378 *A. clavatus* NRRL 1, EED56943 *A. flavus* NRRL3357, EDP48383 *A. fumigatus*, A1163, Q757A5 *A. gossypii* ATCC 10895, GAA86132 *A. kawachii* IFO 4308, KEQ63512 *A*. *melanogenum* CBS 110374, P0C1D2 *A. nidulans* FGSC A4, EMR85414 *B. cinerea* BcDW1, CCU74653 *B. graminis* f. *sp. hordei* DH14, O94030 *C. albicans*, SC5314 Q6FW26 *C. glabrata*, CBS 138 ELA32433 *C. gloeosporioides* Nara gc5 CCT68951 *F. fujikuroi* IMI 58289 Q6CSA1 *K. lactis* NRRL Y1140 EAA30157 *N. crassa* OR74A CDM35704 *P. roqueforti* FM164, CCO31354 *R. solani* AG-1 IB, EDN60085 *S. cerevisiae* YJM789, EEB05198 *S. japonicus* yFS275, NP_593316 *S. pombe* 972h-, EEA22489 *T. marneffei* ATCC 18224, EED15636 *T. stipitatus* ATCC 10500, Q4P8D2 *U. maydis* 521, KDB14263 V. virens; Viridiplantae: JAC74447 *Tetraselmis sp*. GSL018, XP_002269925 *V. vinifera* Metazoa: ACQ58563 A. fimbria, Q6DEI8 *D. rerio*, NP_001091972 *H. sapiens*, ACI69535 *S. salar*. |
| **Erv1** | Fungi: Q9HEK4 *N. crassa*, P27882 *S. cerevisiae*, O14144 *S. pombe*; Metazoa: KFM12471 *A. forsteri*, XP_001120016 *A. mellifera*, KFO95541 *C. anna*, KFO77108 *C. canorus*, JAB87019 *C. capitata*, KFP68003 *C.* *cristata,* ACO10207 C*. rogercresseyi*, EMP36905 Ch. mydas, KFU83859 *Ch. pelagica*, AGB95570 *D. melanogaster*, NP_001082855 *D. rerio*, BAB13348 *E. caballus*, KFP17365 *E. garzetta*, CDS20583 *E. granulosus*, ACO13796 *E. lucius*, CDS26662 *H. microstoma*, P55789 *H. sapiens*, AAG43494 *H. sapiens*, XP_008373045 *M. domestica*, XP_006523576 *M. musculus*, XP_008948643 *M. nubicus*, KFQ31723 *M. unicolo,r* XP_008926462 *M. vitellinus*, ETN76966 *N. americanus*, XP_008854455 *N. galili,* KFR08085 *O. hoazin*, ACO09175 *O. mordax*, XP_003778590 *P. abelii*, KFW96484 *P. carbo*, KFQ61622 *P. crispus*, KFM71151 *S. mimosarum*, XP_008695302 *U. maritimus*; Stramenopiles: CBN79383 *E. siliculosus*; Viridiplantae:EMT33766 *A. tauschii*, NP_564557 *A. thaliana*, 2HJ3 *A. thaliana*, XP_004511174 *C. arietinum*, XP_008454384 *C. melo*, XP_006486938 *C. sinensis*, XP_003542835 *G. max*, EXC05527 *M. notabilis*, AES62810 *M. truncatula*, XP_006651149 O. brachyantha, XP_008792627 *P. dactylifera*, XP_008236433 *P. mume*, EEE87124 *P. trichocarpa*, EEF34893 R. communis, XP_004251228 *S. lycopersicum*, EMS62108 *T. urartu*, XP_002263818 *V. vinifera*, XP_008675741 *Z. mays* |
| **AGK** | Metazoa: JAO92042.1 *P. prolifica,* AAI50066.1 *B. taurus,* NP_060708.1 *H.sapiens* |
| **AIF** | Amoebozoa: XP_636815.1 *D.discoideum,* XP_020436302.1 *P.pallidum*, Metazoa*:* XP_005165182.1 *D.reiro*, NP_112646.1 *R.norvegicus,* NP_004199.1 *H.sapiens.* |
| **Oxa1** | Viridiplantae: Oxa1-1 Q42191 *A.thaliana*, Oxa1-2 NP_182170 *A.thaliana* NP_922381 *O.sativa*; Amoebozoa: XP_004344684.1 *A.castellanii;* EGG16308.1 *P.fasciculatum*; ungy: AAT93151 *S.cerevisiae*; XP_506118 *Y. lipolytica* EAL02067 *C. albicans SC5314;* Euglenozoa XP_001463916.1 *L.iinfantum JPCM5* XP_001681584.1 *L.major strain Friedlin* XP_828689.1*T. brucei TREU927;* Metazoa: NP_005006 *H.sapiens;* NP_081212 *M.musculus;* NP_648417 *D.melanogaster;* EAA11925 *A. gambiae;* XP_537362 *C.familiaris* |
| **Oxa2** | Viridiplantae: Oxa2-1 NP_176688 *A.thaliana*, Oxa2-2 NP_190023 *A.thaliana*; XP_493955 *O.sativa*; Amoebozoa: XP_004344684.1 *A.castellanii;* EGG21561.1 *P.fasciculatum;* Fungy: NP_11576 *S.cerevisiae*; XP_505371 *Y.lipolytica* EAL03076 *C. albicans SC5314;*Euglenozoa: XP_001469267.1 *L.iinfantum JPCM5;* AAK38135 .1 *L.major strain Friedlin* XP_827302.1*T. brucei TREU927;* Metazoa: NP_776188 *H.sapiens* NP_001028482 *M.musculus;* NP_648286 *D.melanogaster;* EAA11323 *A. gambiae;* XP_539312 *C.familiaris* |

**Table S3.** Numbers of exons in genes encoding the predicted subunits for the studied complexes of selected Amoebozoa: *Acanthamoeba castellanii* (*A.c*)*, Dictyostelium discoideum* (*Dd*)*, D. fasciculatum* (*Df*)*. D. purpureum* (*Dp*)*, Polysphondylium pallidum* (*Pp*)*, Entamoeba dispar* (*Ed*)*,* and *E. nuttalli* (*En*), compared to selected representatives of fungi, animals, and plants.

| **Complex** | **Proteins** | **Number of exons** | | | | | | | | | |
| --- | --- | --- | --- | --- | --- | --- | --- | --- | --- | --- | --- |
|  |  | ***S.c*** | ***H.s*** | ***A.t*** | ***A.c*** | ***D.d*** | ***D.f*** | ***D.p*** | ***P.p*** | ***E.d*** | ***E.n*** |
| **TIM9,10** | Tim9 | 1 | 6 | 2 | A:1;B:1;C:3 | 1 | 1 | 1 | 1 | - | - |
|  | Tim10 | 1 | 3 | 5 | A:3; B:2 | 2 | 2 | 2 | 2 | - | - |
| **TIM22** | Tim22 | 1 | 4 | 4 | 6 | 2 | 2 | 2 | 2 | - | - |
|  | Sdh3 | 1 | 7 | 4 | 3 | 3 | 3 | 3 | 3 | - | - |
| **TIM23** | Tim17 | 1 | 6 | 3 | 3 | 1 | 1 | 2 | 1 | - | - |
|  | Tim21 | 1 | 6 | 8 | 4 | 2 | 3 | 2 | 3 | - | - |
|  | Tim23 | 1 | 10 | 1 | 3 | 1 | 2 | 1 | 2 | - | - |
|  | Tim50 | 1 | 12 | 10 | 7 | 5 | 6 | 7 | 6 | - | - |
|  | Mgr2 | 1 | 3 | 3 | 1 | 1 | 2 | 1 | 1 | - | - |
| **PAM** | Pam16 | 1 | 5 | 4 | A:4; B:5 | 2 | 3 | 3 | 4 | - | - |
|  | Pam18 | 1 | 7 | 3 | A:5; B:2 | 2 | 4 | 2 | 2 | - | - |
|  | Tim44 | 1 | 13 | 14 | 4 | 2 | 5 | 2 | 3 | - | - |
|  | mtHsp70 | 1 | 17 | 6 | 3 | 3 | 3 | 2 | 4 | 1 | 1 |
|  | Mge1 | 1 | 4 | 3 | - | 2 | 2 | 3 | 2 | - | - |
|  | Tim15 | 1 | - | ? | 2 | - | - | - | - | - | = |
| **MIA** | Mia40 | 1 | 4 | 4 | - | 1 | 2 | 1 | 4 | - | - |
|  | Erv1 | 1 | 3 | 6 | 1 | 1 | 1 | 1 | 1 | - | - |
|  | AIF | 1 | 18 | - | - | 2 | 7 | 2 | 2 | - | - |
| **OXA** | Oxa1 | 1 | 10 | 9 | 8 | A:2;B:2 | A:2;B:2 | A:1;B:2 | A:2;B:2 | - | - |

**Figure S1.** Alignment of the predicted subunits of the studied complexes displaying differences in amino acid sequences as compared to their counterparts deposited in the GenBank: (A) *Acanthamoeba castellanii* Tim9A; (B) *Polysphondylium pallidum* Tim9; (C) *A. castellanii* Tim10B; (D) *A. castellanii* Tim22; (E) *A. castellanii* Tim50; (F) *A. castellanii* Mgr2; (G) *A. castellanii* Pam16A; (H) *A. castellanii* Pam16B; and (I) *A. castellanii* mtHsp70.

**
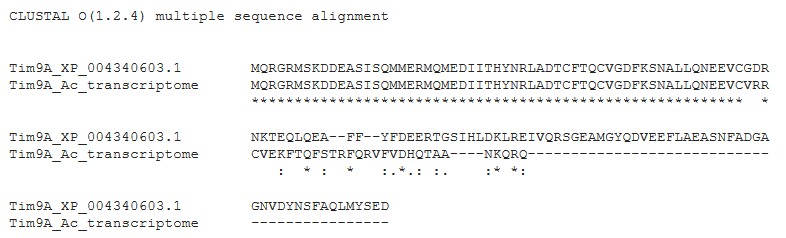
**

**Figure S1A**

**
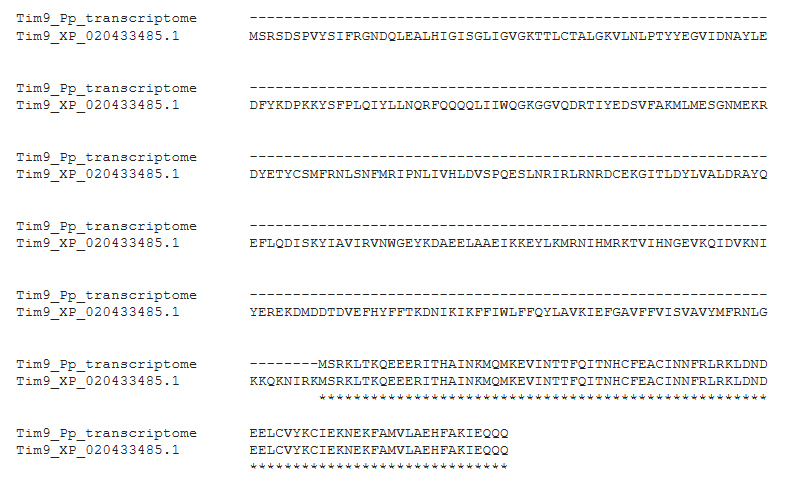
**

**Figure S1B**


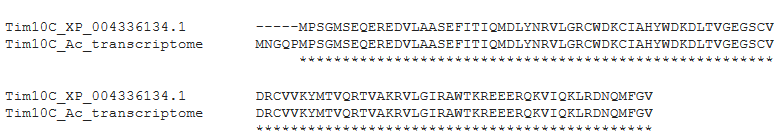


**Figure S1C**

**
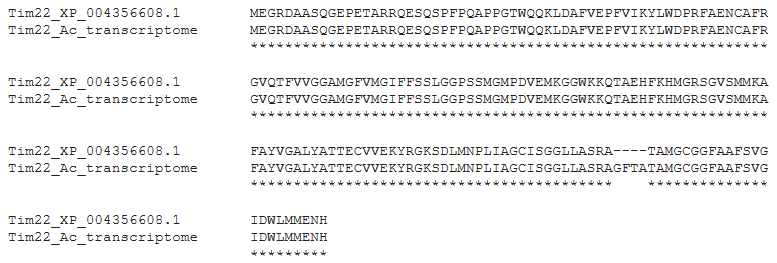
**

**Figure S1D**

**
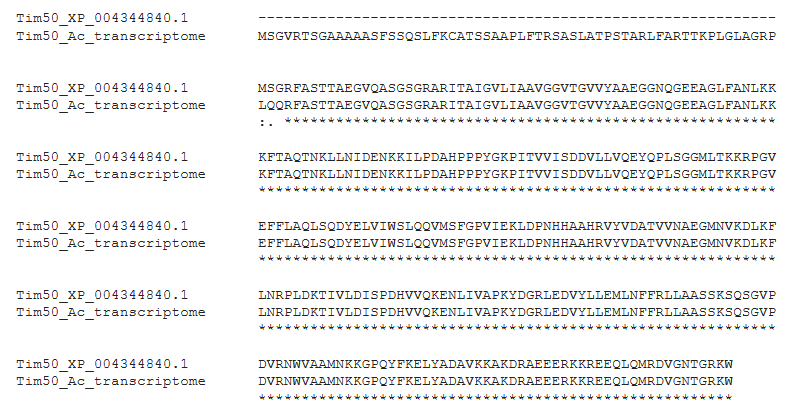
**

**Figure S1E**

**
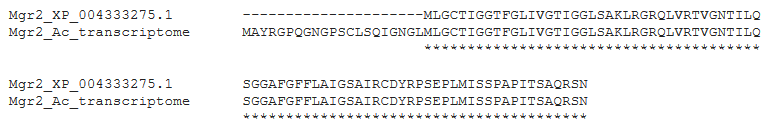
**

**Figure S1F**

**
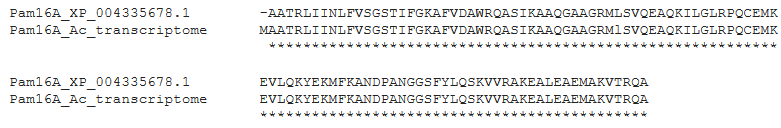
**

**Figure S1G**

**
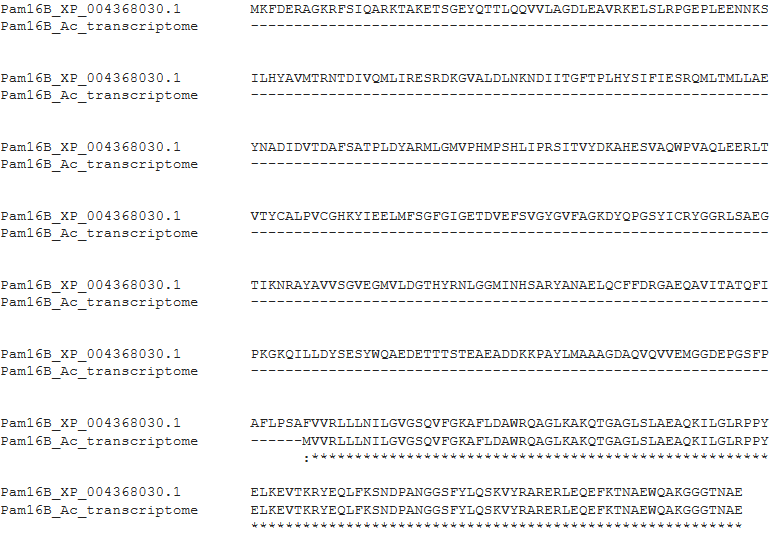
**

**Figure S1H**

**
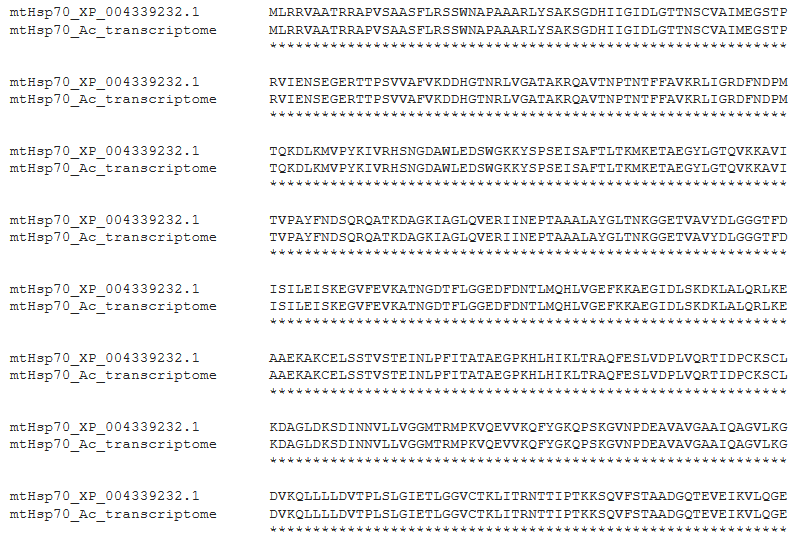
**

**
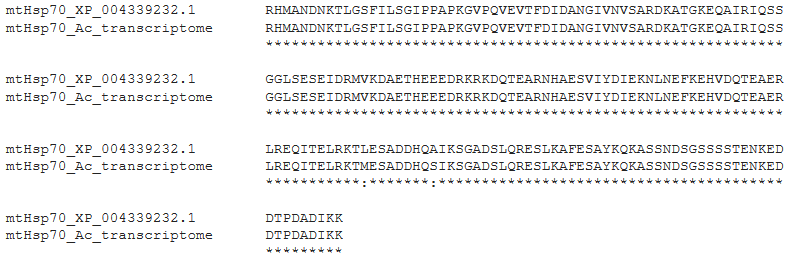
**

**Figure S1I**

**Figure S2.** *Acanthamoeba castellanii* sequences predicted by transcriptome analysis. Amino acid and mRNA assembly correction for the mitochondrial inner membrane and intermembrane space proteins in *A. castellanii* based on transcriptome and genome assemblies.

START and STOP codons are marked with red, while where indicated polyA signals are bolded and underlined.

**1) Tim9A**

Amino acid sequence:

MQRGRMSKDDEASISQMMERMQMEDIITHYNRLADTCFTQCVGDFKSNALLQNEEVCVRRCVEKFTQFSTRFQRVFVDHQTAANKQRQ

cDNA:

AAAAGATCAGAACACAAAGATGCAGCGGGGACGGATGTCGAAGGACGATGAGGCGTCGATCAGCCAGATGATGGAGCGTATGCAGATGGAGGACATCATCACCCACTACAACCGGCTGGCGGACACCTGCTTCACGCAGTGCGTCGGCGATTTCAAATCGAATGCGCTGCTGCAAAACGAAGAGGTCTGCGTGCGGCGTTGTGTAGAAAAATTCACCCAATTCAGCACCCGATTTCAACGCGTCTTCGTCGATCACCAGACGGCAGCCAACAAGCAGCGACAATAGATGTATTCTTTTCTTCATTTTCCTCGAATTCCCCTTTTGTCACTGAGCCTATCCAAGAGCAGAACAGAGATCCGTCGATCGACACAGAAAGAGGAGCATGGGCTATGAACGTCAGATTGAAGCCATCA

Comments: in the NCBI database (XP_004340603.1, ACA1_155100, XM_004340555.1, NW_004457490.1) the protein is mis-annotated. In particular it contains only part of the functional domain of Tim9 proteins, and is fused with EF-hand Ca^2+^-binding domain.

**2) Tim9B**

Amino acid sequence:

MDIAPDAKKSEAAARNMVQLMNEMQMKSVLGMFNSMSERCFRLCMKNVDDDAPITTKEDSCIKNCTEKWQRYSQRVQLIFAEENTRANTKADLMQDRPSSNLEGQD

cDNA:

CCGCAATCGCTCACCAAGATGGACATCGCGCCGGATGCGAAGAAGAGCGAGGCGGCGGCGAGGAACATGGTGCAGTTGATGAACGAAATGCAGATGAAGAGCGTGCTGGGGATGTTCAATTCGATGTCGGAACGATGCTTCCGGCTGTGCATGAAGAACGTCGACGACGACGCGCCCATCACCACCAAGGAGGACTCGTGCATCAAGAACTGCACCGAGAAGTGGCAGCGGTACAGCCAGCGCGTGCAGCTCATCTTCGCCGAGGAGAACACGCGAGCCAACACCAAGGCCGACCTCATGCAAGACCGACCATCATCGAATCTCGAAGGGCAAGATTAACAACAGATTTTTTTCTCAACATCATC

Comments: not present in NCBI database, encoded by a single exon on NW_004457592.1 with start-stop codons at 520329-520008.

**3) Tim10B**

Amino acid sequence:

MNGQPMPSGMSEQEREDVLAASEFITIQMDLYNRVLGRCWDKCIAHYWDKDLTVGEGSCVDRCVVKYMTVQRTVAKRVLGIRAWTKREEERQKVIQKLRDNQMFGV

cDNA:

CGTAACTCAAGATCATGAATGGACAGCCGATGCCCTCGGGGATGAGCGAGCAGGAGCGGGAGGACGTGCTCGCCGCCTCCGAATTCATCACCATACAGATGGACCTTTATAATCGAGTGCTGGGGCGATGTTGGGACAAGTGCATCGCGCACTACTGGGACAAAGACCTTACGGTGGGCGAGGGCTCATGCGTCGATCGATGCGTTGTGAAATACATGACGGTGCAGCGGACAGTGGCCAAGCGAGTGCTGGGCATTCGGGCTTGGACAAAGCGAGAAGAGGAGCGACAGAAGGTTATCCAGAAGCTGAGGGACAATCAGATGTTTGGTGTATAAAATCTTGGCGATTCAAGCAT

Comments: XP_004336134.1 is 5 amino acids shorter. Based on transcriptome assembly, it was decided that Clarke et al. (2013) missed the upstream start codon (ACA1_322180, XM_004336086.1, NW_004457344.1). The version presented here includes these additional amino acids.

**4) Tim22**

Amino acid sequence:

MEGRDAASQGEPETARRQESQSPFPQAPPGTWQQKLDAFVEPFVIKYLWDPRFAENCAFRGVQTFVVGGAMGFVMGIFFSSLGGPSSMGMPDVEMKGGWKKQTAEHFKHMGRSGVSMMKAFAYVGALYATTECVVEKYRGKSDLMNPLIAGCISGGLLASRAGFTATAMGCGGFAAFSVGIDWLMMENH

cDNA:

CTCCTTTCAATTTGGTGATCAAGATGGAGGGCCGGGACGCAGCATCGCAAGGGGAGCCCGAGACGGCTAGGCGACAAGAGTCACAGAGCCCATTCCCACAGGCGCCACCGGGCACATGGCAGCAGAAGCTGGACGCTTTCGTGGAGCCCTTCGTCATCAAGTATCTCTGGGATCCGCGATTTGCCGAAAATTGTGCCTTCCGTGGTGTACAGACCTTCGTTGTTGGTGGCGCGATGGGCTTCGTGATGGGCATCTTTTTCTCGAGCTTGGGTGGCCCCTCCAGCATGGGCATGCCCGACGTTGAGATGAAGGGCGGGTGGAAGAAGCAAACGGCAGAGCATTTCAAGCACATGGGCCGAAGTGGCGTGTCCATGATGAAGGCCTTTGCTTACGTGGGTGCTCTGTACGCCACTACTGAGTGCGTCGTAGAGAAGTACCGGGGCAAGAGTGATCTAATGAACCCGCTCATTGCTGGCTGCATCTCAGGCGGTCTCCTTGCTTCAAGGGCTGGCTTCACAGCAACAGCCATGGGCTGTGGTGGCTTTGCTGCATTTTCTGTTGGCATTGACTGGCTCATGATGGAGAATCACTAGTTGACATAATAACAACTTGACCAAC

Comments: there are two synonymous proteins in the NCBI database: AAT66174 (nucleitode sequence under AY627296.1) and XP_004356608.1 (ACA1_173360, XM_004356555.1, NW_ 004457702.1). The assembled protein sequneces, AAT66174 and XP_004356608.1 are identical except that the later has four amino acids deletion. The corrected version does not contain this deletion.

**5) Tim50**

Amino acid sequence:

MRRIASGTPRSYAAMSGVRTSGAAAAASFSSQSLFKCATSSAAPLFTRSASLATPSTARLFARTTKPLGLAGRPLQQRFASTTAEGVQASGSGRARITAIGVLIAAVGGVTGVVYAAEGGNQGEEAGLFANLKKKFTAQTNKLLNIDENKKILPDAHPPPYGKPITVVISDDVLLVQEYQPLSGGMLTKKRPGVEFFLAQLSQDYELVIWSLQQVMSFGPVIEKLDPNHHAAHRVYVDATVVNAEGMNVKDLKFLNRPLDKTIVLDISPDHVVQKENLIVAPKYDGRLEDVYLLEMLNFFRLLAASSKSQSGVPDVRNWVAAMNKKGPQYFKELYADAVKKAKDRAEEERKKREEQLQMRDVGNTGRKW

cDNA:

CTCGAATCTTGAGGTGGGGCACATTTTCTGCATCTGCGCATCTCGATCTACGCGTATATCTACAGAAGGCAAGCTACGATGAGGCGAATCGCATCAGGCACTCCACGTAGCTACGCTGCGATGTCAGGCGTGCGGACCTCCGGCGCAGCGGCAGCGGCTTCATTCTCATCTCAATCGCTCTTTAAGTGCGCCACATCATCTGCTGCCCCTCTTTTCACCCGCAGCGCTTCCCTCGCAACTCCTTCCACAGCACGGCTCTTCGCTCGCACGACCAAACCCCTCGGTCTTGCGGGCCGGCCCCTGCAGCAGAGATTCGCCTCGACCACCGCCGAGGGCGTCCAAGCTTCGGGCAGCGGCAGGGCACGTATCACCGCTATTGGCGTACTCATTGCTGCCGTTGGTGGCGTCACCGGTGTGGTCTACGCTGCTGAGGGTGGCAACCAGGGCGAGGAGGCTGGCCTCTTTGCCAACCTCAAGAAGAAGTTCACGGCTCAGACGAACAAGCTGCTCAACATCGACGAGAACAAGAAGATCTTGCCAGACGCTCACCCTCCTCCGTATGGCAAGCCCATCACAGTGGTCATCTCGGACGACGTCCTGCTCGTGCAAGAATATCAGCCGCTCTCCGGCGGTATGCTGACGAAGAAGCGCCCTGGCGTTGAGTTCTTCCTGGCCCAGCTCAGCCAAGACTACGAGCTCGTCATCTGGAGCTTGCAGCAAGTCATGAGCTTTGGCCCCGTCATCGAAAAGCTGGACCCGAACCATCACGCGGCGCACCGGGTCTACGTCGACGCTACGGTGGTCAATGCCGAGGGCATGAACGTGAAGGACCTCAAGTTCCTCAACAGACCATTGGACAAGACGATCGTTTTGGACATCAGCCCGGATCACGTGGTGCAGAAGGAGAACCTCATCGTGGCCCCTAAGTACGACGGCAGGCTCGAAGACGTCTACCTCTTGGAGATGCTCAACTTCTTCCGGCTGTTGGCCGCCTCGTCCAAGTCGCAGAGCGGAGTGCCCGATGTGCGCAACTGGGTGGCGGCCATGAACAAGAAAGGCCCGCAGTACTTCAAGGAGCTGTACGCCGACGCTGTGAAGAAGGCCAAGGACAGGGCCGAGGAGGAGAGGAAGAAGAGGGAGGAGCAGCTTCAGATGCGTGACGTCGGCAACACCGGCAGGAAGTGGTAACGAAGCATTGTTGCGTTCTTGTTGAGCTCTCTTCGTGAAGGGCGAACAGCGAGCCGTCGTGTAGCGTG

Comments: XP_004344840.1 in NCBI database is shorter as the part of the encoding gene first exon is excluded from the respective mRNA (ACA1_282730, XM_004344790.1, NW_004457586). Start-codon/exon-end coordinates of the first exon are 104865/725994. The coordinates of the consecutive exons are the same as in the NCBI database.

**6) Mgr2**

Amino acid sequence:

MAYRGPQGNGPSCLSQIGNGLMLGCTIGGTFGLIVGTIGGLSAKLRGRQLVRTVGNTILQSGGAFGFFLAIGSAIRCDYRPSEPLMISSPAPITSAQRSN

cDNA:

GGAAAACCGGTGAGGTGGGAGGGCGAAGAAAGCCAATCTAATTTTGCATCTTCAGACGACACATCGAACAGTTTAATTCAAATCTACATCAATATGGCATACCGAGGACCGCAAGGCAATGGCCCGTCGTGCTTGTCGCAGATTGGAAACGGCCTGATGCTGGGATGCACGATTGGCGGCACGTTCGGTCTCATCGTGGGCACCATTGGTGGACTGTCGGCCAAGCTGCGAGGACGGCAGCTCGTGCGGACGGTGGGCAACACCATCCTGCAGAGCGGCGGCGCCTTCGGATTCTTCCTCGCCATCGGCAGCGCGATTCGATGCGATTACCGACCGAGCGAGCCGCTGATGATTTCTTCCCCCGCTCCCATCACCTCTGCGCAACGCTCCAACTGATTGTCAACGATCGCCCCAACAATACCAACAAAATCAA**ATTAAA**CATTCCAAAAAAA

Comments: XP_004333275.1 in NCBI database is shorter as the ATG codon - 63 bp upstream of the recognized in this version ATG - is missed in the respective mRNA (ACA1_127430, XM_004333227.1, NW_004457261.1).

**7) Pam16A**

Amino acid sequence:

MAATRLIINLFVSGSTIFGKAFVDAWRQASIKAAQGAAGRMlSVQEAQKILGLRPQCEMKEVLQKYEKMFKANDPANGGSFYLQSKVVRAKEALEAEMAKVTRQA

cDNA:

GGTGGATCGTCGTTCACGACAGGAATCTTGAACGCATCGGTCAACGACACCTTTCTCTTTTCTACAATACACCACCCATGGCGGCGACAAGATTAATCATCAATCTTTTTGTCTCGGGCAGCACCATCTTCGGTAAGGCCTTTGTCGATGCGTGGCGGCAAGCGAGCATCAAAGCGGCGCAAGGTGCTGCCGGCCGTATGTTGAGTGTTCAGGAGGCGCAAAAGATCTTGGGGCTGCGACCACAGTGCGAGATGAAGGAAGTCCTGCAGAAATATGAGAAAATGTTCAAGGCCAACGATCCTGCGAACGGAGGATCCTTCTACTTGCAGTCCAAGGTGGTGCGCGCCAAGGAGGCGCTGGAGGCCGAGATGGCCAAGGTCACACGGCAAGCCTAAGCGGTGTGCCCTTCCTAATGATGGAAATCACAAATCGTTGTACATATCCGGCCCTTTTTTTTACCGCAAAGACAGCCTGCATTCATCGCTCCATCCATCCACTGC

Comments: XP_004335678.1 in NCBI lacks Met at N-terminus, that is also reflected in errors in the respective mRNA (ACA1_038500, XM_004335630, NW_004457329.1). The corrected assembly version of the gene contains additional upstream exon at coordinates 165850-165910. Only one codon – that is ATG start codon - is being translated in the amino acid sequence.

**8) Pam16B**

Amino acid sequence:

MVVRLLLNILGVGSQVFGKAFLDAWRQAGLKAKQTGAGLSLAEAQKILGLRPPYELKEVTKRYEQLFKSNDPANGGSFYLQSKVYRARERLEQEFKTNAEWQAKGGGTNAE

cDNA:

TTCCGATCTCCCAGATCAAACATGGTGGTGCGACTGCTCTTGAACATCCTGGGCGTGGGCTCGCAAGTGTTCGGGAAGGCCTTCCTCGATGCCTGGCGACAAGCCGGCCTCAAGGCCAAGCAGACCGGGGCAGGCCTCTCGCTGGCGGAGGCGCAGAAAATTTTGGGGCTGAGACCGCCCTACGAACTCAAGGAAGTCACCAAGCGATACGAACAGCTGTTCAAGTCAAACGATCCTGCCAATGGTGGGTCCTTCTATCTGCAGTCCAAGGTCTACAGGGCACGAGAACGACTGGAGCAGGAGTTCAAGACCAACGCGGAGTGGCAGGCCAAGGGCGGCGGCACCAACGCCGAGTGACAACTGCCAGCATCGCCACACCGTCACACACCAGCAGCAAAAAAGGAGTCTCTCAACTTATTCACACAACACCCCTTCGCTCACCTGACCTGCGGCGAGAGTCAAGAAGAGAT

Comments: XP_004368030.1 in NCBI database seems to represent erroneously fused genes (ACA1_290500, XM_004367973, NW_004457712.1). The corrected cDNA includes 4 exons, while the NCBI-stored version - 13 exons. The additional 9 exons encode ankyrin domain and SET domain.

**(9) Pam18B**

Amino acid sequence:

MQRTTAQAAKTSQPKMGPGTIFLALGVGGAGLALAPRLVSSAAKHFSKGGSSGKGFATMGSMWKRSGGGASTGFKNFYKGGFESEMTRAEAALILGIRQSAPKEKIRLAHRRIMLLNHPDNGGSDYMASKINEAKDVLVKDLN

cDNA:

CCGGTTCAAGAAGAATCAGAGCACCACCGCGCACGCACAGAAAGAAGACCGCAACAAGTCAGAAACAGTATGCAGAGAACAACAGCACAGGCCGCGAAGACGTCCCAGCCGAAGATGGGACCGGGCACAATCTTCCTCGCGCTGGGCGTGGGCGGCGCTGGACTTGCGCTGGCCCCGCGACTGGTCTCATCGGCCGCCAAGCATTTCTCCAAGGGCGGCAGCTCTGGCAAGGGCTTCGCGACGATGGGATCGATGTGGAAGCGCTCGGGAGGGGGCGCCAGCACGGGCTTCAAAAACTTCTATAAGGGCGGATTCGAAAGCGAGATGACCAGAGCGGAGGCTGCCCTAATCTTGGGCATCAGACAGAGTGCACCCAAGGAAAAGATCAGGCTGGCGCATCGACGAATCATGTTGCTCAACCACCCAGATAATGGCGGCTCCGATTACATGGCCTCGAAGATCAACGAGGCCAAGGACGTTCTCGTCAAAGATCTCAATTAGCTTCTTTCGCCCGTTGATCGCTGAGCCCGACGGCAGTGGGTCCACCAGAGGAAAGAGTTTTCTCACCCACGCGGTCATGAGCAGCGCTGCATCTTCTCACATCA**AATACA**AATGTCTATTTACACTCGCAAAAAAAAAAA

Comments: not annotated in NCBI database. The transcript is, probably, represented by two exons on NW_004457253.1. The coordinates for the first exon (1-362 bp in the presented here cDNA) are at coordinates 54-415 on the contig, start codon is at 346. The existence of the both exons, however, are supported by WGS sequences (blastn search against traces WGS for *A. castellanii*): accessions numbers for the best matches are ti:2136034119 and ti:2139207236.

**(10) mtHsp70**

Amino acid sequence:

MLRRVAATRRAPVSAASFLRSSWNAPAAARLYSAKSGDHIIGIDLGTTNSCVAIMEGSTPRVIENSEGERTTPSVVAFVKDDHGTNRLVGATAKRQAVTNPTNTFFAVKRLIGRDFNDPMTQKDLKMVPYKIVRHSNGDAWLEDSWGKKYSPSEISAFTLTKMKETAEGYLGTQVKKAVITVPAYFNDSQRQATKDAGKIAGLQVERIINEPTAAALAYGLTNKGGETVAVYDLGGGTFDISILEISKEGVFEVKATNGDTFLGGEDFDNTLMQHLVGEFKKAEGIDLSKDKLALQRLKEAAEKAKCELSSTVSTEINLPFITATAEGPKHLHIKLTRAQFESLVDPLVQRTIDPCKSCLKDAGLDKSDINNVLLVGGMTRMPKVQEVVKQFYGKQPSKGVNPDEAVAVGAAIQAGVLKGDVKQLLLLDVTPLSLGIETLGGVCTKLITRNTTIPTKKSQVFSTAADGQTEVEIKVLQGERHMANDNKTLGSFILSGIPPAPKGVPQVEVTFDIDANGIVNVSARDKATGKEQAIRIQSSGGLSESEIDRMVKDAETHEEEDRKRKDQTEARNHAESVIYDIEKNLNEFKEHVDQTEAERLREQITELRKTMESADDHQSIKSGADSLQRESLKAFESAYKQKASSNDSGSSSSTENKEDDTPDADIKK

cDNA:

CGGATCCGGCACGAATCTTTAGATCTCAACCCTCTCACTACACAACACAAGCATCACGATGCTCCGCCGCGTTGCTGCCACTCGTCGTGCCCCAGTCTCTGCTGCCTCCTTCCTGAGGAGCTCATGGAACGCCCCTGCCGCTGCCAGGCTCTACTCTGCCAAGAGCGGTGATCACATCATCGGTATCGATCTCGGTACCACCAACTCGTGCGTGGCCATCATGGAGGGCTCTACGCCTCGCGTGATCGAGAACTCGGAGGGTGAGCGCACCACTCCCTCGGTGGTGGCCTTCGTCAAGGACGACCACGGCACCAACCGTCTGGTCGGCGCCACTGCCAAGCGTCAGGCCGTGACCAACCCCACCAACACCTTCTTCGCCGTCAAGCGTCTCATCGGCCGCGATTTCAACGACCCGATGACCCAGAAGGATCTCAAGATGGTGCCCTACAAGATTGTCCGTCACTCGAACGGCGATGCCTGGCTCGAGGACAGCTGGGGCAAGAAGTACTCGCCCAGCGAGATCAGCGCCTTCACCCTCACCAAGATGAAGGAGACCGCGGAGGGCTACCTCGGCACCCAGGTCAAGAAGGCCGTCATCACCGTGCCCGCCTATTTCAACGATTCTCAGCGTCAGGCCACCAAGGACGCCGGCAAGATCGCCGGTCTCCAGGTTGAGCGTATCATCAACGAGCCCACTGCCGCCGCCCTCGCCTACGGTCTTACCAACAAGGGTGGCGAGACCGTCGCCGTCTACGATCTCGGTGGTGGTACCTTCGATATCTCCATTCTCGAGATCTCCAAGGAGGGTGTCTTCGAGGTGAAGGCCACCAACGGTGATACTTTCCTCGGCGGTGAGGATTTCGATAACACTCTCATGCAGCACCTCGTGGGCGAGTTCAAGAAGGCCGAGGGCATCGATCTCTCCAAGGACAAGCTCGCTCTCCAGAGGCTCAAGGAGGCTGCCGAGAAGGCCAAGTGTGAGCTCTCTTCCACCGTGTCGACCGAGATCAACTTGCCCTTCATCACTGCCACCGCTGAGGGTCCCAAGCACCTCCACATCAAGCTCACCCGCGCCCAGTTCGAGTCGCTCGTCGATCCCCTCGTCCAGCGCACCATCGATCCCTGCAAGTCCTGCCTCAAGGATGCCGGCCTCGATAAGTCCGACATCAACAACGTCCTGCTCGTCGGCGGCATGACCCGTATGCCCAAGGTCCAGGAGGTCGTCAAGCAGTTCTACGGCAAGCAGCCCAGCAAGGGTGTCAACCCCGATGAGGCCGTCGCCGTCGGTGCCGCCATCCAGGCTGGCGTGCTCAAGGGTGACGTCAAGCAGCTCCTGCTGCTCGACGTGACGCCCCTCTCGCTCGGCATTGAGACTCTCGGTGGCGTGTGCACCAAGCTCATCACCAGGAACACCACCATCCCCACCAAGAAGTCCCAGGTCTTCTCCACCGCCGCCGACGGCCAGACCGAGGTCGAGATCAAGGTCCTCCAGGGCGAGCGCCACATGGCCAACGACAACAAGACCCTCGGCTCCTTCATCCTCTCCGGCATCCCGCCCGCCCCCAAGGGCGTGCCCCAGGTCGAGGTCACCTTCGACATCGACGCCAACGGCATCGTCAACGTCTCCGCCCGCGACAAGGCCACCGGCAAGGAGCAGGCCATCCGCATCCAGTCCTCCGGCGGTCTCTCTGAGTCGGAGATCGACCGCATGGTAAAGGACGCCGAGACCCACGAGGAGGAGGACCGCAAGCGTAAGGACCAGACCGAGGCCCGCAACCACGCCGAGTCCGTCATCTACGACATCGAGAAGAACCTCAACGAGTTCAAGGAGCACGTCGACCAGACTGAGGCCGAGCGTCTGCGCGAGCAGATCACCGAGCTCAGGAAGACCATGGAGTCGGCCGACGACCACCAGTCCATCAAGTCCGGCGCCGATTCGCTCCAGCGCGAGTCCCTCAAGGCCTTCGAGTCGGCCTACAAGCAGAAGGCCTCGTCCAACGACAGCGGCAGCAGCAGCAGCACCGAGAACAAGGAGGACGACACCCCCGACGCCGACATCAAGAAGTAGACAACCAGCCACCCCCCCAACAACAACCAACAACCGACATTCCTCCAGCCAATCCTCCAACCCTCCCCACCTCTCTTGTTTATAGTGCGGCGCGGAAGAGCCTCCGCCCCTAGCTAAACCCCCCCAAAACAAAGACAGTTTTGATCTAAAAAAAA

Comments: related IDs from NCBI database: XP_004339232.1, ACA1_058850, XM_004339184.1, NW_004457442.1. Several nucleotides in the NCBI-annotated version were corrected. Referring to the gene sequence, the corrections are at: 394586 ncbiC->corA that results in Ala->Ser; 394610 ncbiA->T that results in Leu->Met.

**11) Mge1**

Amino acid sequence:

MRAAVRRLPNVASHSAASRGIFALNVPLRSRTLAIARPFSTEQNGEQKVEGEHPPAQQQAEGENKEQQQEQQDPQAAKIDELQNKVKDLEEQNKELNGNYLRSLADLDNMARIGKTNVENAKLYSIKSFAEGMLEIADNLSRALESLPEEKRGLPDVKVLFEGVAMTERVLQQVFARYGIKKFNPLNEKFDPTKSSALFEIQDPTKAPGTVAFVQAPGYTLHDRLLRAAQVGVVAQPPDEPSSSDQA

cDNA:

CCGGGCCACGCCGAGCTCAAAATTCGTTAATCGCGACCTTGGCATTCATCACCCGATCAACCACAGTACACACCCACACCTACACACAAGATGAGAGCCGCCGTCCGCAGATTGCCCAACGTCGCCTCACACTCGGCCGCCAGCCGGGGCATCTTCGCCTTGAATGTGCCGCTCCGCTCCCGAACACTTGCCATCGCTCGTCCCTTCTCCACTGAGCAAAATGGGGAGCAGAAGGTCGAGGGTGAGCATCCCCCTGCTCAGCAGCAGGCCGAAGGAGAGAACAAGGAGCAGCAGCAGGAGCAGCAGGATCCTCAGGCGGCCAAGATTGATGAGCTGCAGAATAAGGTCAAGGACCTGGAGGAGCAGAACAAGGAGCTGAACGGCAACTATCTTCGATCGCTCGCCGATCTCGACAACATGGCACGCATCGGCAAGACCAACGTCGAGAACGCCAAGTTGTATAGCATCAAGAGCTTCGCCGAGGGCATGCTCGAGATTGCGGACAATCTCAGCCGAGCTCTCGAATCGCTGCCCGAGGAGAAGCGAGGGCTGCCCGACGTGAAGGTGCTCTTCGAGGGCGTCGCCATGACCGAGCGCGTGCTTCAGCAGGTGTTCGCCCGCTACGGCATCAAGAAGTTCAACCCCCTCAACGAGAAGTTCGATCCCACCAAGAGCTCGGCCCTCTTCGAGATTCAGGACCCCACCAAGGCGCCCGGCACTGTCGCCTTCGTGCAGGCGCCCGGCTACACCCTCCACGACCGTCTCCTCCGAGCGGCGCAGGTGGGCGTCGTCGCTCAGCCCCCCGACGAGCCATCATCATCTGACCAGGCCTAAAGATCAAAAAAGAGAAAAA**AATAAAATAAA**AAAAGATTAAATGCCAAA

Comments: this protein is not present at NCBI database but it is supported by the hits found in blastn alignment against WGS (traces) *of A. castallanii* genome (ti:2134983330). This gene has two overlapping potential polyA signals.

The amino acid sequences for **Tim9C**, **Tim10A, Sdh3, Tim17, Tim21, Tim23, Pam18A, Tim44, Erv1** and **Oxa1** are identical between the transcriptome assembly and the genome assembly and did not require any correction. Here only the nucleotide sequences are presented as the cDNA sequences from Clark et al. (2013) do not feature UTR regions included in the study.

**1) Tim9C**

cDNA:

TCTATCCATCCATCAAAGAAGTGAGCATGGGAAACAGACAGTTCGGGATGGGCTACGGAATGGGTGCCGGCCAGTCCCGAGCGGGATCGGCATCGGATGAGGAGCGGATGATGCAGGTCATGATGGCCTCCATGCAGATGCAGGACTATCTCACAATGTACAACTCTACCACGGAGAAGTGCTTCAAGAAGTGCGTCTTCAACCTCCGCACCCCACAGCTCGTTGAGAAGGAAGAGGTCTGCCTCAATCGATGCATCGAAAAGATGTCCCACTACAACCTGCGATTTCAACAGAAGGTGGGCGCGGAGAGCGCGGCGTTGCGCGAGCAGCAGCAGAAGGAAGCCGAGCAGAAGCAGCAGCCACCAACCGAAGCCGCCCAGGCCGACAAAGAAGCCCCATCAAGCAAGTAACGGACTC**AATACA**AACTCACTTCACACCACAAAAAAA

**2) Tim10A**

cDNA:

CGAAGAGACAACAGTTTCGATCAAGATGTCGAACGAGCAGTACATGACGCGGGAACAGATGCTGGCTTCGGCACAGCAGGAGCTCGTTGCCTTCCAGGACATGTACAACAGAATGCAGCACCAGTGCTGGAAGAAGTGCGTATTGCGTTTGGGTGAGTCGGAGCTGAGCGTGTCGGAAGGTCTTTGCGCTGATCGATGCGTCAAGAAGTACATGGAGGTGCACAACCGCGTGGGCAAGGTGCTGCAGGGGCTGCAGCAGCAACAGCCGCAACCGCAGTAAGAAACGCGAGTGGGCAAAAACCAAACACTTCAAGGCCAACCACGAGATGTGGTTGCTACGCCTGTGTGAAAGTAGGGGCACAGAACACTGCAAAGAGTG

**3) Sdh3**

cDNA:

GTCAGATTTTGCGTCATTGATTCAACAATCGATTGTTAATTAGCCAAGAGGAAAGAAAATGCTGAGGCAGGCCACCGCTCGCACCGCTCCCCTGTTCGCCGTACGCGGCACCGTCACAGGCCTTCAGGTCCTCAGCCGCTCGCCGGTGGCGCTGGCGACTCGCACCCTCGCCACCCTCCCCTCCGATGCCTCGACGGAGGCCAAGCCCAACAGGCCTCTTTCGCCCCACGTCTCCATCTACAGGTTCCCTCTGCCCGCCTTGACCTCGATCACCAACCGCGCCACTGGTGGTGCCCTCACCGCCGGTATCTACACTGCTGGTGCCCTCGCTCTCTTCGGTGCTCACGATCTTCCCGTCTACATCGACGCCTTCAAGGCTGCCGTGCCCCTCCTTGTCTACCCCACCAAGCTCCTCGTCTCTTTCCCCTTCGTCTACCACACGCTGGCCGGCATCAGGCACTTGTACTGGGACTACACGGCCAAGGGACTCACCCTCCCCGAGGTCTACACCTCGAGCTACGCGCTCATGGGCGCCACGGCTCTGCTCACCCTCGGCCTCACCTTCTACTCGATCTGATCGTCTCGCTCATGATGACGGCCGACCGCCGTTCCTTCTCTTCGGCGTTTGTGGTGCTGCTAGTTGTTCAC**GATAAA**CGTCGTACCTCTATTTCTAAAAAAAAA

**4) Pam18A**

cDNA:

CCGGAACTTGCATCTGCGACCATCTGCCGCTGTATCGATCCGGGAGCA(ATT)GGCTACTTTCTTGTGGCGGGTGTGGTGATTGCTGGTGTGGCCATCGGTGGGCGCGTGGCTATGAACGCCTACAAGCACTTCAAGGCCGGCAACTTGACCCTCCCCAAGGGCATGGTGCCCAAGGGTCCATCGAGGATGAAGTCCTACTACACGGGCGGCTTTGAGTCGGAGATGACCCGCGCCGAAGCCGCTCTCATCCTCAGTGTCCGACAAGGCGCCTCGAAGGAGAAGATCAAGATGGCCCACAGGCGGATCATGTTGGCGAACCATCCCGACAATGGAGGCAGCGACTACGTGGCGTCGAAGGTGAACGAAGCCAAGGACCTGCTGCTCAAGGATCTCGGCGACGACTGAGGCCCCTCTTCCAAAATAACGAGAGCACCAAAAGGACCACCCCCACCAAACACAGACACACACCGAGACACCACACACCGCCGCCGCCGCCGCCACCAGAGAGATCGGGGCTGTACAGAGTACTACACCACCATATATGCACACCCATATTACCAACAAAAAAA

**4)Sdh3**

cDNA:

GTCAGATTTTGCGTCATTGATTCAACAATCGATTGTTAATTAGCCAAGAGGAAAGAAAATGCTGAGGCAGGCCACCGCTCGCACCGCTCCCCTGTTCGCCGTACGCGGCACCGTCACAGGCCTTCAGGTCCTCAGCCGCTCGCCGGTGGCGCTGGCGACTCGCACCCTCGCCACCCTCCCCTCCGATGCCTCGACGGAGGCCAAGCCCAACAGGCCTCTTTCGCCCCACGTCTCCATCTACAGGTTCCCTCTGCCCGCCTTGACCTCGATCACCAACCGCGCCACTGGTGGTGCCCTCACCGCCGGTATCTACACTGCTGGTGCCCTCGCTCTCTTCGGTGCTCACGATCTTCCCGTCTACATCGACGCCTTCAAGGCTGCCGTGCCCCTCCTTGTCTACCCCACCAAGCTCCTCGTCTCTTTCCCCTTCGTCTACCACACGCTGGCCGGCATCAGGCACTTGTACTGGGACTACACGGCCAAGGGACTCACCCTCCCCGAGGTCTACACCTCGAGCTACGCGCTCATGGGCGCCACGGCTCTGCTCACCCTCGGCCTCACCTTCTACTCGATCTGATCGTCTCGCTCATGATGACGGCCGACCGCCGTTCCTTCTCTTCGGCGTTTGTGGTGCTGCTAGTTGTTCAC**GATAAA**CGTCGTACCTCTATTTCTAAAAAAAAA

**5) Tim17**

cDNA:

CGGCGAGCAACACGGCACACATCAATCGTTGAATTGATTCATCACACACAAGAGAGACACACAAATCAATGGACGCCTACAGAGATCCCTGCCCTTCCCGTATCGTCACAGACTGCGGCAGCGCGTTCGCCATGGGCGCAATCGGCGGCAGCCTTTGGCATGGCGTCATCATGGGCTGGCCCCAAGCGCCACGAGGGATGCGCATGTCCTCCGCCATTACGGCTCTCAAAACGAAGGCGCCGAGCCTGGGCGGCAGCTTCGCCGTGTGGGGCGGCCTGTACTCCAGCTTCGATTGCACATTCGCCTATCTGCGAGGAAAGGAAGACTTCAAGAACTCCATCATGTCGGGTGCTGCCACCGGTGCGGTCCTCGCTGCGCGAACGGGATGGAAGGGATCTCTGAAGAGCGGTGTGGTGGGGGGTGGCCTGCTGGCGTTGATCGAGGGTATTGTCTTCGTCATCTCGCGACAAAGCCCAGCTCAGCAGCAAGATTTCGGCTACGCGCCGCCACCCCCGCCGCTCGAGGATGTGCTCGATGTGGAGGAGAAGGGCGGTTTCTTCTCCAGCCTCACCAACCTCTTCTCGAAGAAGGAGGAATCGAAACCCTTCGAGATGGAAGAGGAGACGACGATGGACGATTTCGGTCAGGAGACGAAGGAGGACGATTTTGGCGATCTCTTCCAGCCACCGCCCGCCACCTCCTGGGGCGACGACAGCTGGAACACAGAAGAGAAGGAACACTCCTAAGCCCTCTCTTCTTCTACTACTCCTTCCTTTACTACACTAAAACTCCCACTCCACAAACCAAAAAAAA

**6) Tim21**

cDNA:

AAGAAATACGCAAAGAAAAACATCACGTAGAGATCGAGCAAAATGCAGCGATCTGCCATCTGCACCAGCGGGCGTGCGTTGTTGGGCGGCCGCGTGTCTCTTGCCACGGCATGCACCCGGCCAGTGGGCATCGTCGGCCGGCGTGGGGTGTGGGAGGGCGCCCTCTATTCTCGGCCACAGCCGCAGCAGCAGAGTCTTGGGAGACGGAGGTTCGCGACCGACTCGAACGGGGGCCAAAAGGAGCCTCCCTCGGGCACCAAGGGGGGCCTGCAGGCCGCCAAACCCTCCGGCCAGGGCACCGCCGTCACCGTCGCGCAAAAGGTGGTGGAGGGGGGCAAGGACGCGGGGTACGGGCTGGTGATCCTGGGCGGCGTGGTGCTGCTGGGCGCGGCGGCCTACCAGCTGTTCTCGGCCGGGTTTGCGCCGTCGAGTCCGCAGCACGTGTACTCCAAGGCGTCGGATCTTCTCCGCCGCGACCCGGAGGTGCAGAAGCTGCTGGGTCCCAACATCAAAACCTACGGCGAGGAGACCAGCCGACGGCGCCGCGGCCTCCAGTCGACCAAGTACTTCAACGCCGCCACCCAGCGCGACCACGTGCGAGTACTCTTCAGCGCCGAGGGAGACGTCAACGCCGCCGACGTCATCGCCGACGTCACCGCCTCGGGCCAATTCTACCTCCTCACCGTCGAGGTGCCCGTCACTGGAGAGAAGCTGGTGTACACCAACACCGGGAAGTTCGAGCGCCGCTGATGGGTGGGGGCGGCCCACCAATACCACTTCCGGACGGAGAGAGAGAGAGAGGAGCGAAGAGCCCATGCAGGGGGGAGGGGCAATGCTGCACGTGCACCGGCGCCTTTCTCTCTCCCCACCCGAGAGCGAGCCCCGGGAAGAGACCAGAGACATTTGAAAGAGAGGCGCAGAGCCACGTTCAGGCATGGTTCGGCGCCTTTACGCACTTTGTACAGGAAGGAGGAGGAGGCCCAGACGGGGGAGATTTGGGGGGGGGGGAAGGAAGCAAGGTTCACCTGCGCCACGACGACGACGTGGGTCAGGTCTCGATGCGCATGGGGTCGGGGCCTG

**7) Tim23**

cDNA:

ACGAGATCTGCAGAGATTTTGTAATCGATTGGAGCGGCACGAAACAGCGGCATCATATTAGCAGAACACACTACAATGGGTGACTCCTCGTACGCCGACTCTTCTTCCATGCCCGATTTTGATGCGTTCCCAGAGCCCAAGCTGGACAACATCGGCATCGATGTCGATTCGATTAGCTACACAAATCCGATGTATGGACTCTCCGGACCCAAGGGCGCCGAATACATCTTCGCGGACGACAACCAGGCCCGAAAGATGTCCTGGGCTGATCGATCAACTTGGCTCTGGGGTGGTGCCTGGCTCACCGGTGGAGTGATCGGCACGACATGGGGTGGATTGAAGGGTCTGCGAGATGCCGAGCGTGGTCTGCCGTGGAAGCTGCGATTAAACGCCTGCCTCAACGGTGCCGGCCGAAAGGGTGGCCGCATGGCCAACGGCCTCGGCGTGTTGGTGCTTCTCTACTCGGGCGTCGAGACGGCCACTGCGGCCGCGAGGCAGAAGCAAGATGGTCTCAACATCGTCGCTGGCACCACCATGGCGCCTCTCATCTACTGGAGTGGAGCCGGCCTCATCAGGAGCATGGCGTTCGGCGCGCTTGGTGGCGTTGTTGGCTTCGGGCTTGTCCTGGGTCACAAGTACAACGTGATGGGCCTGCGAAAGATCCTTCCCGCCGACGAGTAAAACCACTTCTCCTCTCCACACACACACACACACCTCCACCACCTTTACTTCATTCTCCTCCCACTTCAATCACCTTCGCCCTCTACTTCTTCTCCTCTTCTTCCGTACACACCTCGACGTCTTCGGCATCAACTTCTCTCTTCACTTGCTCCTCTGCAGCTTCTCCAACCTCTCTGGTTATGACCAGCAGGCGAGGTCTGGCTGCAGCAGAGGGTCTCTCCTCTTCTCCTTGCACTCCTACCCTCTTGTATTTATTTGTCTCTCTTTTGGGCTGGTCTGCAGCCACACCCACACCATCAACCTTTACACACAACACTACAAGATAAATAC**AATACA**AGATCATGTTTGCAACAAAAAAAA

**8) Tim44**

cDNA:

AGCTTTTGGAGCGAATTCGTCAAGTCCTTCAAGGAGCAGGCGGAGAACAGCAAGGAGCTGAACGAGTCGATGGCCGAGCTCAAGAAGCTTCAGGAGTCAGAAAGCCTGAAAAAGACGAAGGAGTCAGTGGAAAAGCTGAAGGACAAGTCGGCGGAGGGGCTCGCGCAACTGAAGAACCTGGGCGAAAAGGCGGCGGCGACCGGGGGACGTGTGACAGCACGGGTGTCGGACTCAGTGACATCGACCCTGGGCGACATCACGCAGCGTGTGGGCATGGACGAAAAGCTGGAGAAGACGAAAGAGGGCTTCGAGAAGGTGGCCGATAGCTTAAACAAGGCGCGGGAGAACATCGAGAAGGTGGGAGAGAACATCAAGACCTCGGCCGAAGAATCGGAGCTGGTCAAGAAGGTCAAGGAGAAGGTGCCGTTCGGCCTCCTCGACGGATCCGATGCGGCCGCCGGCGAAGGGAGTGGCGGCGAGGGCGCAGTCGCTGGAGCCCGGTCGCAGCTCGTGGTCAAGACCCAGGAAGTGCAGACCGCCATCGAGAAGCGTCTTCAGGACGCTCGCGACCGGTTGCGAGCCACGGGCGCCTTCAAGAAGGCTCTCGCTCTGCGCGAGAAGATCGTAGAAAGCGATAACCCACTCATCCAACGCGTGCTCGACCTTGGCGATGGCATCACGCGCACCACCTCGAAGCTCTTCTCCGAGAACGACCACGCCAAGACGATCAAGCTCATCCACGAGCTCGATCCTACCTTCAGTCCGGAGAAGTTCAAACAGAAGATGGCCTCCTCCTTTGTGCCCACCCTCCGCTCGGCTCTGCTCCGCGGCGATCCCGCACCCATCTCTGCCTTAATGTCCAAGAGGCTGGCTGAGCACGAGCTAGAGGCCTACAAGGAGTGGGACGCCCACGGCTATCGCTCCCATTCCAAGCTCCTGAATCTTGACACTGTCGAGGTGCGCCTCTCTCCCCCCCCCACTGCTTTCTCTCCTTTGTGCTGCTCTGTACTAACACCGCCCCATCTCATTCCCCGCTCCTGTGCAGATCCTCAATGCCCATACAAGGGATGATGGACGGCCAACACTGCTCATCCACTTTGTGTCCCAAGAGAACCACTGCATCCGTGATAGTTCAGGCGCTGTGGTTGAAGGCGCTCCGGTGCGCTTTATTCCTTCCTTGCACCTTTCACACGTTTGCTGACCTAGCCTGTCTCTCTGTTTTCCAGGACAATATCCAGGATGTTGAGCAAACATGGGCCATGCAGATCAACAGCCAGGGAGAATGGGAAGTTATTGAGTACATGGTGGTAGCTTCTAGGCCATCCTGGTAG

**9) Erv1**

cDNA:

ATGGAGCAATTCAAGAAGATTAAGGAGAAGCTGCAGAAAGAGGGTGATCAACTCGTGGTGGGTGGTGCAGATGAAGAAGATTGTGGTGCATGTAAGCCACGAGCGGGCGGACTCTACGGCAGCGACATGCGCAGAAGGTTCCGCTCACAGCAGCAAGCACGAGAACAGAGATCGGCTCAGGAGCAAGATGGCGGCACGACGTCAACTGACGCCACGGCAACATCTGCCGGCACAGAAGAGGATGCATCGAATGAGAACACCACCGAGTGCTACGACAGCGAGGGCTACTGGGAGAAGCAGGACGCACCCGATGTGATCGAGCTGGGCCAGGCAGGCTGGACGCTACTCCACAGCATGGCGGCTTACTACCCGCCCAAGCCGTCGCGGCAGCACCAAGAGCGCATGCGCAGCTTCCTCTCCCTGTTCCCTCACCTCTACCCCTGCAAGGTGTGCGCCAAAGACTTCGAGGAGACGATGGACGAGATCCCGCCCGAGCTCGAATCCCACGATGCGCTCTCGGACTGGATGTGCCGCGCGCACAATCGAGTAAATCAGCAACTGGGCAAGCCGCTCTTCCCCTGCGAGCGCGTACGCGAGCGGTGGGGTCCATCCAATGCATTCCACACCAAAGAGCAAGATGATTCGTAG

**10) Oxa 1**

cDNA:

ATGCTCATTCGGCATGAGCGGAGGTGCTACGCCACCGAGACTGTCGAGGTATTCCCCCAAAGCTTGATGGATTGAATCAAATTCAATTTTTTAAAAAATAATGAACGATGATGATCGCACACAGGAGGGCGCTACTGCAGCGGCCGAGGCGGCGACACAGACGGGCGCGGCGGTGGAACGCGGCAGCGATGCGATGGCCTCGTTCATCGCGGCCGACCCGGTGACCAGCTTGACGCCGGCAGCGCTGGAGGTGGTGGAGCGCACGACGAGCTCGTTCCCGCCCATCAACTGGCTCGTGGACGCCATGACGTTCGTCCACTACACCGTGCCTCTCTCGTGGGCCGCGACCGCCGTCTGCTTCACCCTCGCCTTCCGTACCATGATCTTGCCTGCCGTACGCCATCCGAGTGGTCCCTTGACATTCTCCTGATGACGCTCACCTTGTCGTGTGCGTGTGTCATGTTCAGGTGGCCCTGACGATGCAAAACAGCGCCAAGATGCGCGCCGTTCAGCCCGGTACACACGGCACACACACACGACACACACGACACACACGACACACACGACACACACACACGCACCACACACACACACACACGACACCACACACACACGACACACACACGCACCACACACACACACACCACACACACACACGACGCACACAGTGTAAGCTTTTTGTTTACATCCGTGCGGTGTCGACCACAGAGCTGGAGAAGATCAAGGCCAAGTACGGCCCCAACGTCGGCAAGGACGTCAACGTCGCCATGCAGTACCGCGAGGAGGTACGGCTGTGGCCCCACCACAAACCCAACGCGCCTCATACCCGCCTTGACCGCCCTTCCGCGTCCATGCGCGTGCTGGTCGCAGGTGAGGAAGATGATGGCCCAGTCCGGCGTCAGCATGTGGAAGACGATGGTGCCCTTCCTCGTGCAGACGCCCCTGTTCGTCAGCTTCTTCTTCACCACCAGGAAACTTGGTACGCGCTAACGCTCATTCATTTTCATTCATAAATAATTCGTTGCGCTGACCCCTGGCACTTGTAGCTGAGGAGGAGCCGGGACTTCGGGATGCCAGCTTCCTGTTCTTCAACGATCTGTCGGCGGCGGATCCCTACTACATCATGCCCATCCTCACTTCGCTCACCATGCTCGCCACCATCGAGGTACGCAGCGCGCCCCCGAGCCATACAGTGACGCAACAGTTCGCGCTCTACGCGGCCAACTGACGCACAATACTCCCTCGGGTATGTGTATGTGTATGTGGAACACACAGCTGGGCTCTGATGGTGTCGGCGGTCAGAGCAACCCGGCGCTCAAGAACTTCCTGCGACTCTTCTCCGTGCTCGCCATTCCGGCCACGTCCAGCCTTCCCATCGTACGCGCCCCTCTTGTCCCTTGGATCGACGTCGTGTAGCTTGCTGACGTGCGACCACTTCGCGTTTTCTCTCCTACGCGGCGGCAACAACCAGTTCACGCACATCTACTGGTTCTCGTCGAACTTGTTTTCGCTGTGCCAGCTGGGCCTGTTCAAGGTGCCCTTCATCAAGAAGGCCCTCGGCCTGCCCGACTACCAGGGCGTGTCGCCCATGCTGGGTAGCTCGGACCCGCAGCCCGCCAAGCCCGAGGTGACCTTCCAGCAAAAGCCCAGGATCATCAGGACCAAGAACCAGAAGTAG

**11) Tim15**

cDNA:

ATGCAACGTACCCTCCCTCCTTCCTTGTTCTGTATTGCATTATTGCTTATTCTAGTATTATTTAGCTTAATTAATTGACATCAATTCTGATGAGCAGGTAGGGTAGCAACAGTTGCAAAGGGGGAGCTGGCCCTGCTGGCGCGCTCGGTGCCAAGTTCTGCACTTCTGGGTATACGCCAGTCTACTTTGGCCTCTCGCGTATCAGCTTCGCCGCTGCACGGTGCTGCTACCAGGCCACGACCATGGTCGCCCTCCTGCTTCCTTCGCATCAGCGCCCGATCGCTCCACGCCTCTTCCACGCTGCAGCGCAGCGCCAAGTCGAGCATGGTGGAGGCTACCTCTGCCGATGACCCCACAGACAGCGCCGAGGCCAAGGAGACCAGCGCTGAGTCGGCTGCTACGCCGACGACCACAACGACGCTGAACATCGGACGCATACAGCCCAAGCTCGAGCTGCACTACACGTGCGGCGTGTGCGAGCTGCGCAGTACGAAGCAGTTCAGTCGCGTGGCCTACGAGAAGGGCGTGGTCATCATCCGCTGCGGCGGGTGCGAGAGCCTCCACCTGATCTCGGACAACCTGGGCTGGTTTGGCGAGGACAAGAACATCGAGGAGATCATGCGCAAGCGCGGCGAGGCCGTCGAGCGAGGGCGCAGGGACGCCGGTGGCAACATTCTCCTCGACAACAACAACACCAATACCGCCGGCGACGATACCATCACCACCGACCTGGAGCACGACGGCACCCAGACAGTCGTCGAAGAGTCCGAAGACTGCATCCTCATCACCCCTACGGCCGCAGTAAGGTAG
